# Single-cell profiling reveals an endothelium-mediated immunomodulatory pathway in the eye choroid

**DOI:** 10.1084/jem.20190730

**Published:** 2020-03-20

**Authors:** Guillermo L. Lehmann, Christin Hanke-Gogokhia, Yang Hu, Rohan Bareja, Zelda Salfati, Michael Ginsberg, Daniel J. Nolan, Santiago P. Mendez-Huergo, Tomas Dalotto-Moreno, Alexandre Wojcinski, Francisca Ochoa, Shemin Zeng, Juan P. Cerliani, Lampros Panagis, Patrick J. Zager, Robert F. Mullins, Shuntaro Ogura, Gerard A. Lutty, Jakyung Bang, Jonathan H. Zippin, Carmelo Romano, Gabriel A. Rabinovich, Olivier Elemento, Alexandra L. Joyner, Shahin Rafii, Enrique Rodriguez-Boulan, Ignacio Benedicto

**Affiliations:** 1 Department of Ophthalmology, Margaret Dyson Vision Research Institute, Weill Cornell Medicine, New York, NY; 2 Regeneron Pharmaceuticals, Inc., Tarrytown, NY; 3 Caryl and Israel Englander Institute for Precision Medicine, Department of Physiology and Biophysics, Weill Cornell Medicine, New York, NY; 4 Angiocrine Bioscience, Inc., San Diego, CA; 5 Laboratorio de Inmunopatología, Instituto de Biología y Medicina Experimental, Consejo Nacional de Investigaciones Científicas y Técnicas, Buenos Aires, Argentina; 6 Developmental Biology Program, Memorial Sloan-Kettering Cancer Center, New York, NY; 7 The University of Iowa Institute for Vision Research and Department of Ophthalmology and Visual Sciences, The University of Iowa, Iowa City, IA; 8 Wilmer Ophthalmological Institute, Johns Hopkins Hospital, Baltimore, MD; 9 Department of Dermatology, Weill Cornell Medicine and New York-Presbyterian Hospital, New York, NY; 10 Departamento de Química Biológica, Facultad de Ciencias Exactas y Naturales, Universidad de Buenos Aires, Buenos Aires, Argentina; 11 Ansary Stem Cell Institute, Department of Medicine, Division of Regenerative Medicine, Weill Cornell Medicine, New York, NY; 12 Centro Nacional de Investigaciones Cardiovasculares (CNIC), Madrid, Spain

## Abstract

The activity and survival of retinal photoreceptors depend on support functions performed by the retinal pigment epithelium (RPE) and on oxygen and nutrients delivered by blood vessels in the underlying choroid. By combining single-cell and bulk RNA sequencing, we categorized mouse RPE/choroid cell types and characterized the tissue-specific transcriptomic features of choroidal endothelial cells. We found that choroidal endothelium adjacent to the RPE expresses high levels of Indian Hedgehog and identified its downstream target as stromal GLI1^+^ mesenchymal stem cell–like cells. In vivo genetic impairment of Hedgehog signaling induced significant loss of choroidal mast cells, as well as an altered inflammatory response and exacerbated visual function defects after retinal damage. Our studies reveal the cellular and molecular landscape of adult RPE/choroid and uncover a Hedgehog-regulated choroidal immunomodulatory signaling circuit. These results open new avenues for the study and treatment of retinal vascular diseases and choroid-related inflammatory blinding disorders.

## Teaser

The authors report the transcriptome of mouse retinal pigment epithelium/choroid tissue at single-cell resolution. A subset of choroidal endothelial cells expresses Indian Hedgehog, which targets mesenchymal stem cell–like cells regulating mast cell survival and the inflammatory response after damage.

## Graphical Abstract

**Figure:**
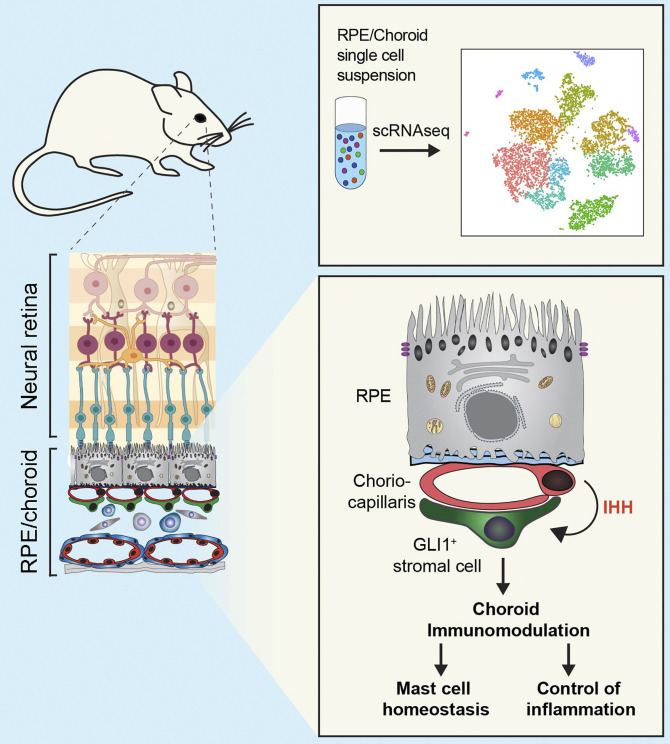

## Introduction

Photoreceptors capture incoming photons and transform them into electrical pulses that ultimately lead to visual perception. Their localization to the outer retina, distal to the incoming light and visual processing neurons of the inner retina, is a successful evolutionary design that allows vertebrate photoreceptors to benefit from the critical support of two external eye layers, the retinal pigment epithelium (RPE) and the choroidal blood vessels. The RPE enables photoreceptor function by eliminating stray light, phagocytosing photoreceptor waste products necessary for their renewal, recycling visual cycle components, and constituting an essential component of the outer blood–retinal barrier between choroidal circulation and the neural retina (Strauss, 2005). Whereas separate retinal blood vessels nourish the inner retina, blood supplied by the choroidal circulation is the main source of oxygen and nutrients for RPE and photoreceptors and the main evacuation route for retinal waste (Nickla and Wallman, 2010). Thus, choroidal perfusion is essential for RPE and retinal homeostasis. However, this is likely not the only role of choroidal endothelial cells (ECs), as recent studies have shown that microvascular ECs are not passive conduits for delivering blood but rather organ-specific factories of highly specialized sets of angiocrine factors that regulate tissue homeostasis and regeneration (Rafii et al., 2016). Recently, we provided initial evidence for this scenario in the eye. We reported that developing and adult mouse choroidal ECs exhibit different transcriptomes, and such transition is likely required for the coordinated establishment of the outer blood–retinal barrier and the acquisition of visual function (Benedicto et al., 2017). However, specific transcriptome features of choroidal ECs compared with retinal and extraocular ECs and the molecular identities of nonendothelial choroidal cell types have not been determined in detail so far. RPE and choroid act as a functional unit, and defects in any of their supportive roles can cause retinal degeneration and blinding diseases such as age-related macular degeneration (AMD). AMD, an incurable disease that affects 8.7% of the worldwide population and 25% people >80 yr old, is characterized by photoreceptor loss secondary to dysfunction or death of the RPE and choroidal ECs (Ambati and Fowler, 2012; Mullins et al., 2011; Seddon et al., 2016; Wong et al., 2014). The etiology of AMD remains largely unknown, in part due to the very limited availability of molecular information about the different cell types that populate the choroid and the intercellular networks that maintain RPE/choroid tissue in a healthy and functional state.

Single-cell RNA sequencing (scRNAseq) is a recently developed approach that allows efficient transcriptional profiling of individual cells in a given tissue or other complex cellular populations (Macosko et al., 2015). This technology has opened the door to answering longstanding biological questions on tissue cell diversity, heterogeneity of cellular responses, and regulatory signaling networks that would be difficult or impossible to answer using traditional physiological or biochemical approaches. A recent landmark study characterized by scRNAseq the molecular identity of all cell types in the mouse neural retina but did not analyze RPE or choroid (Macosko et al., 2015). More recently, scRNAseq was used to create a mouse cell atlas covering all major organs, but RPE and choroid were not included (Han et al., 2018). Here, we report a scRNAseq analysis of adult mouse RPE/choroid and provide the transcriptional signature of choroidal ECs compared with other tissue-specific ECs, including retinal ECs. Our studies uncover the molecular identity of the major choroidal cell types, including three different EC subtypes and a previously uncharacterized population of mesenchymal stem cell (MSC)–like cells. Moreover, MSC-like cells are the target of EC-secreted Indian Hedgehog (IHH), which was found to be a critical modulator of choroidal and retinal inflammatory responses. These results open new avenues for the study and treatment of retinal vascular diseases and choroid-related inflammatory blinding disorders such as AMD.

## Results

### Characterization of mouse RPE/choroid by scRNAseq

**Figure 1.**
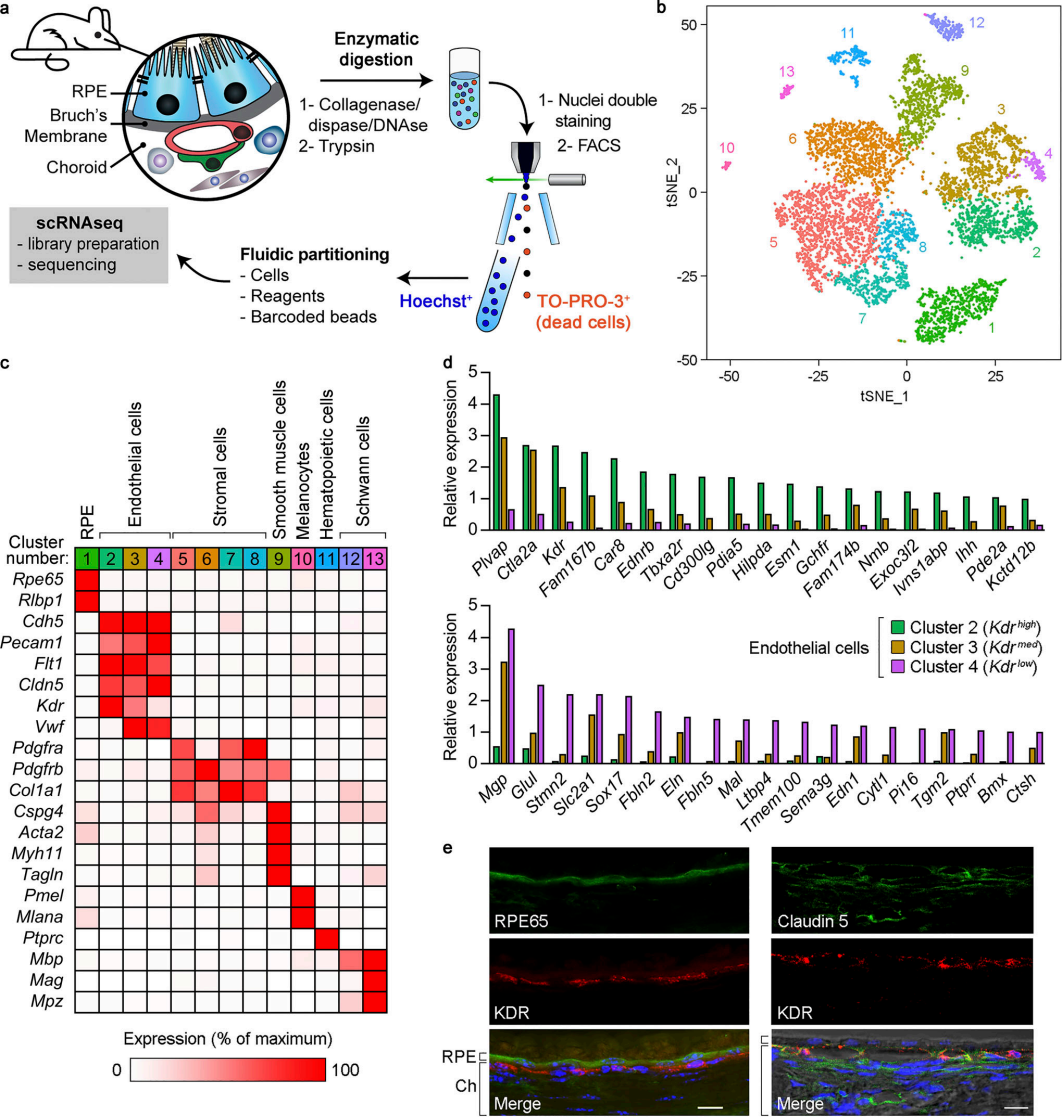
**Characterization of mouse RPE/choroid by scRNAseq and identification of transcriptionally distinct choroidal EC subtypes.** **(a)** Scheme of the preparation of RPE/choroid single-cell suspensions by sequential enzymatic digestion and cell sorting. Hoechst^+^/TO-PRO-3^−^ live cells were subjected to scRNAseq. **(b)** Principal-component analysis of 7,723 single-cell expression profiles obtained from RPE/choroid tissue from four mice (C57BL/6J and B6129PF1/J strains, one male and one female per strain). Data are shown in two dimensions using tSNE. Unsupervised analysis clustered cells into 13 transcriptionally distinct cell populations, each plotted in a different color. **(c)** Identification of cell types in RPE/choroid tissue from B6129PF1/J mice according to the average expression of known markers in each cluster. Cluster number colors are the same as in panel b. Relative gene expression among cell types was calculated using the average normalized UMIs in each cluster and represented as the percentage of the cluster with maximum expression. White, 0%; red, 100%. **(d)** Genes with at least fivefold differential expression between *Kdr^high^* (green bars) and *Kdr^low^* (purple bars) ECs (Benjamini–Hochberg adjusted likelihood-ratio test, adj P < 0.05). Only genes with maximum average normalized UMIs ≥1 are shown. Relative gene expression in *Kdr^med^* ECs (brown bars) is also included. Top: *Kdr^high^* expression at least fivefold higher than *Kdr^low^*. Bottom, *Kdr^low^* expression at least fivefold higher than *Kdr^high^*. **(e)** Immunofluorescence analysis of KDR (red), RPE65 (RPE marker), and claudin-5 (pan-endothelial marker; green) expression in mouse eye cryosections. Nuclear staining with DAPI (blue) and differential interference contrast images are also included in merged panels. Position of RPE and choroid (Ch) is indicated on the left of merged images. Scale bars, 5 µm (left) and 10 µm (right). Images are representative of four mice.

**Figure S1.**
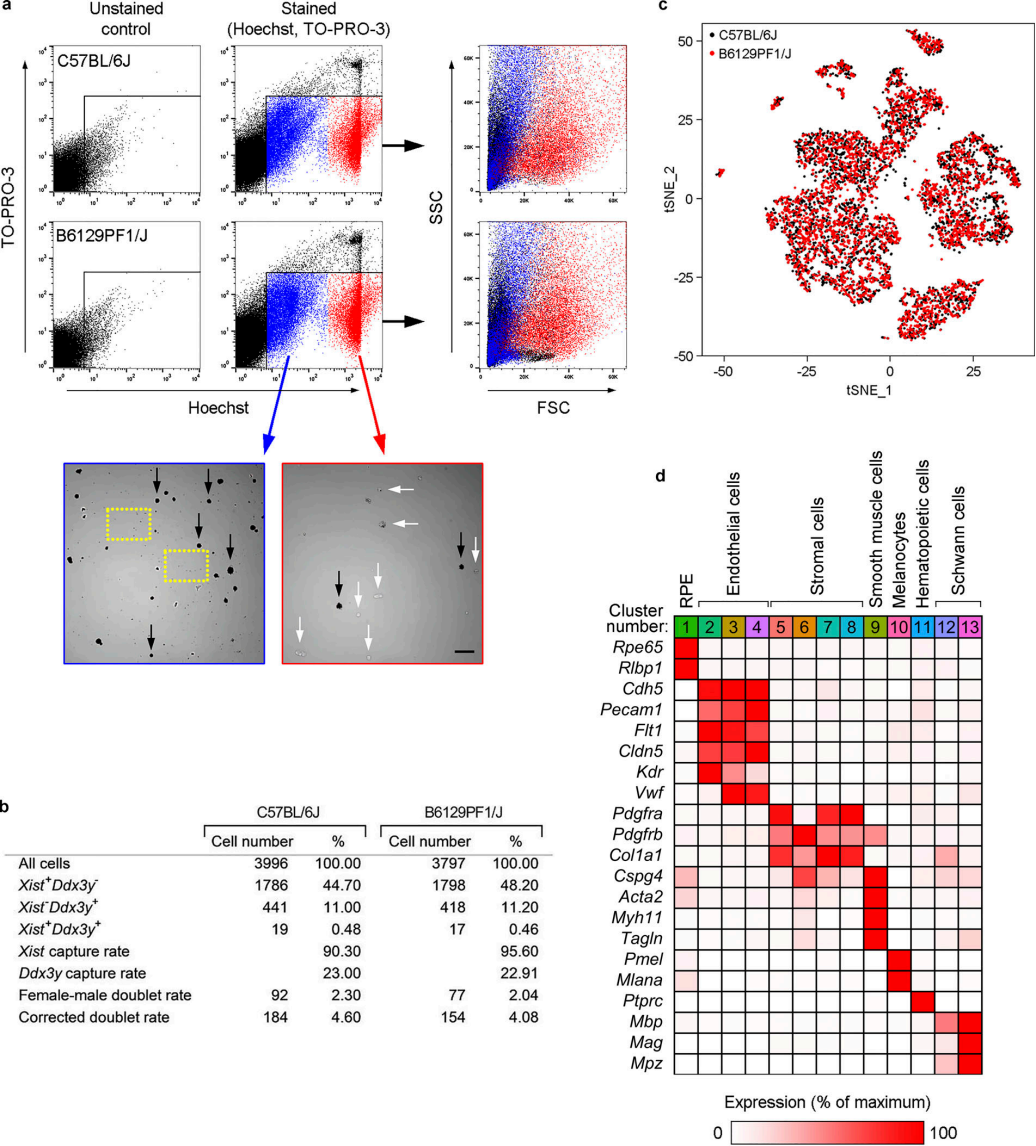
**scRNAseq of RPE/choroid tissue.** **(a)** Cell sorting to prepare RPE/choroid single-cell suspensions suitable for scRNAseq. Experiments were performed with the strains C57BL/6J (top panels) and B6129PF1/J (bottom panels). For each strain, RPE/choroid tissue from one male and one female eye was pooled, digested, and stained with Hoechst and TO-PRO-3 to label nucleated and dead cells, respectively. Unstained samples were used as controls to set the gating parameters. Pilot experiments showed two different Hoechst^+^/TO-PRO-3^−^ populations with dim (blue dots) and high (red dots) Hoechst staining. Hoechst^dim^ events presented very low forward scatter (FSC; right panels), suggesting that this population could be debris. To assess the cellular content of both populations, we sorted Hoechst^dim^/TO-PRO-3^−^ and Hoechst^high^/TO-PRO-3^−^ events independently and observed them under the microscope. The Hoechst^dim^ population contained cell debris (dashed yellow squares) but also many cells that were mainly pigmented (black arrows). Hence, our results suggest that cell pigment present in RPE and melanocytes may decrease the apparent FSC of both cell types. The Hoechst^high^ population (red) was virtually free of debris and mainly constituted by nonpigmented cells (white arrows). To avoid loss of pigmented cells, both Hoechst^dim^/TO-PRO-3^−^ and Hoechst^high^/TO-PRO-3^−^ populations were sorted together in the same collecting tube and used for scRNAseq assays. Scale bar, 50 µm. **(b)** Estimation of doublet content in scRNAseq assays (see calculations in Materials and methods). **(c)** Principal-component analysis of 7,723 single-cell expression profiles obtained from a mix of male (*n* = 1 per strain) and female (*n* = 1 per strain) RPE/choroid tissue. Data are shown in two dimensions using tSNE after CCA batch correction. Black dots, 3,996 cells from C57BL/6J mice; red dots, 3,727 cells from B6129PF1/J mice. **(d)** Identification of cell types in RPE/choroid tissue from C57BL/6J mice according to the average expression of known markers in each cluster. Cluster number colors are the same as in Fig. 1 (b and c). Relative gene expression among cell types was calculated using the average normalized UMIs in each cluster and represented as the percentage of the cluster with maximum expression. White, 0%; red, 100%.

Transcriptional profiling at single-cell resolution requires high-quality cell suspensions. To this end, we developed a fast and efficient procedure to isolate single cells from RPE/choroid by sequential tissue digestion and cell sorting. We used FACS to optimize the capture of single, viable cells (TO-PRO-3 negative) and separate nucleated cells (Hoechst positive) from cell debris (Figs. 1 a and Fig. S1 a). We surgically dissected RPE/choroid tissue from eyes enucleated from 90-d-old mice of two different strains, C57BL/6J and B6129PF1/J, and sequenced 3,996 and 3,727 cells from each strain, respectively. To avoid any potential sex-related bias during our analyses, we pooled tissue from two eyes, one male and one female, for each strain during enzymatic digestion. In addition, this approach allowed us to estimate that ∼4% of isolated cells were present as doublets (i.e., two cells barcoded with the same sequence), based on the assumption that male–female doublets accounted for 50% of total doublets (Fig. S1 b; Macosko et al., 2015). Unsupervised clustering of individual cell transcriptomes based on similarity in overall gene expression identified 13 transcriptionally distinct clusters within RPE/choroid cells (Fig. 1 b), with remarkably similar results for both strains (Fig. S1 c). Using the FindConservedMarkers function in Seurat, we found ≥65 conserved cluster-specific genes for each cluster in both mouse strains (adjusted [adj] P < 0.05; Data S1 a). Based on these gene lists and the expression of known cell-type–specific markers, we categorized these clusters as RPE, ECs, stromal cells, smooth muscle cells, melanocytes, hematopoietic cells, and Schwann cells (Fig. 1 c, B6129PF1/J strain; Fig. S1 d, C57BL/6J strain; and Table 1). This analysis identified subtypes within ECs (clusters 2–4), stromal cells (clusters 5–8), and Schwann cells (clusters 12 and 13). The complete datasets for both strains are reported in Data S1 (b and c).

**Table 1.** Mouse RPE/choroid cell types identified by scRNAseq

Cluster number	Cell type	Cell number		Percentage of total		Average UMIs/cell		Average genes/cell		Average UMIs/gene	
		C57BL/6J mice	B6129PF1/J mice	C57BL/6J mice	B6129PF1/J mice	C57BL/6J mice	B6129PF1/J mice	C57BL/6J mice	B6129PF1/J mice	C57BL/6J mice	B6129PF1/J mice
1	RPE	402	445	10.1	11.9	4,860	7,514	1,468	1,911	3.02	3.51
2	ECs	405	366	10.1	9.8	4,550	5,151	1,920	2,090	2.18	2.32
3	ECs	550	435	13.8	11.7	4,847	5,570	1,943	2,139	2.28	2.42
4	ECs	74	63	1.9	1.7	4,749	5,196	1,894	2,025	2.26	2.37
5	Stromal cells	776	797	19.4	21.4	3,255	3,723	1,524	1,645	2.09	2.21
6	Stromal cells	610	552	15.3	14.8	3,171	3,796	1,491	1,681	2.07	2.21
7	Stromal cells	223	229	5.6	6.1	3,695	4,518	1,684	1,894	2.14	2.32
8	Stromal cells	158	166	4.0	4.5	2,995	3,944	1,391	1,661	2.13	2.33
9	Smooth muscle cells	476	400	11.9	10.7	3,863	4,769	1,583	1,814	2.38	2.58
10	Melanocytes	28	20	0.7	0.5	3,913	5,008	1,621	1,847	2.32	2.59
11	Hematopoietic cells	140	116	3.5	3.1	4,573	4,960	1,528	1,496	2.83	3.19
12	Schwann cells	108	106	2.7	2.8	2,525	2,835	1,306	1,418	1.90	1.96
13	Schwann cells	46	32	1.2	0.9	2,606	3,532	1,227	1,495	2.08	2.31
	Total	3,996	3,727								

Data are from C57BL/6J and B6129PF1/J mice (pooled cells from one male and one female for each strain).

### Identification of transcriptionally distinct choroidal EC subtypes

The choroidal vasculature plays key roles in retinal homeostasis and in the pathogenesis of blinding diseases such as AMD (Bhutto and Lutty, 2012; Nickla and Wallman, 2010; Whitmore et al., 2015); hence, we were particularly interested in further characterizing choroidal EC subtypes. EC clusters 2–4 presented similar expression levels of the generic EC markers *Cdh5* (VE-cadherin), *Pecam1* (CD31), *Flt1* (VEGFR1), and *Cldn5* (claudin-5); however, they displayed remarkable variations in the expression of other markers. Because *Kdr* (VEGFR2) was expressed at decreasing levels in EC clusters 2–4 (Fig. 1 c and Fig. S1 d), we termed these clusters *Kdr^high^*, *Kdr^med^*, and *Kdr^low^* ECs, respectively. To study the most significant transcriptional differences between *Kdr^high^* and *Kdr^low^* ECs, we selected the genes whose expression in *Kdr ^high^* was detected by an average of ≥1 normalized unique molecular identifier (UMI), and the average number of normalized UMIs was at least fivefold higher than in *Kdr^low^* ECs and vice versa. Average normalized UMIs for each gene in *Kdr^med^* were also included in the analysis for comparison purposes (Data S1, d and e). Using this filtering approach, we found 19 genes specifically enriched in *Kdr^high^* compared with *Kdr^low^* (Fig. 1 d, top panel) and 19 genes with the opposite expression pattern (Fig. 1 d, bottom panel). In general, gene expression in *Kdr^med^* was intermediate between *Kdr^high^* and *Kdr^low^*, suggesting that *Kdr^med^* constitutes a transitional EC population between the other two.

The choriocapillaris is the capillary network immediately adjacent to the RPE basement membrane that provides nutrition and a waste evacuation route to the outer retina, and it is characterized histologically by the presence of fenestrations in the EC plasma membrane (Lutty et al., 2010; Nickla and Wallman, 2010). As the gene with highest expression in *Kdr^high^* ECs is *Plvap* (Fig. 1 d, top panel), which encodes the fenestrae-associated protein PV1 (Stan et al., 2012), it is likely that the *Kdr^high^* cluster represents choriocapillaris ECs, whereas the *Kdr^low^* cluster corresponds to ECs from deeper choroid capillaries. To test this hypothesis, we performed immunofluorescence experiments using antibodies against KDR and RPE65, an RPE marker. KDR was detected in the innermost choroidal layer very close to the RPE (Fig. 1 e, left), consistent with the location of the choriocapillaris. We also costained KDR and claudin-5, a pan-endothelial marker expressed at similar levels in *Kdr^high^*, *Kdr^med^*, and *Kdr^low^* choroidal ECs (Fig. 1 c and Fig. S1 d). Claudin-5 expression was found both in KDR^+^ ECs and in deeper choroidal layers closer to the sclera with undetectable KDR expression (Fig. 1 e, right), the latter likely corresponding to *Kdr^low^* ECs. These experiments strongly suggest that *Kdr^high^* and *Kdr^low^* clusters correspond to transcriptionally and geographically distinct populations of choroidal ECs.

### Tissue-specific enrichment of *Ihh* expression in choriocapillaris ECs

**Figure 2.**
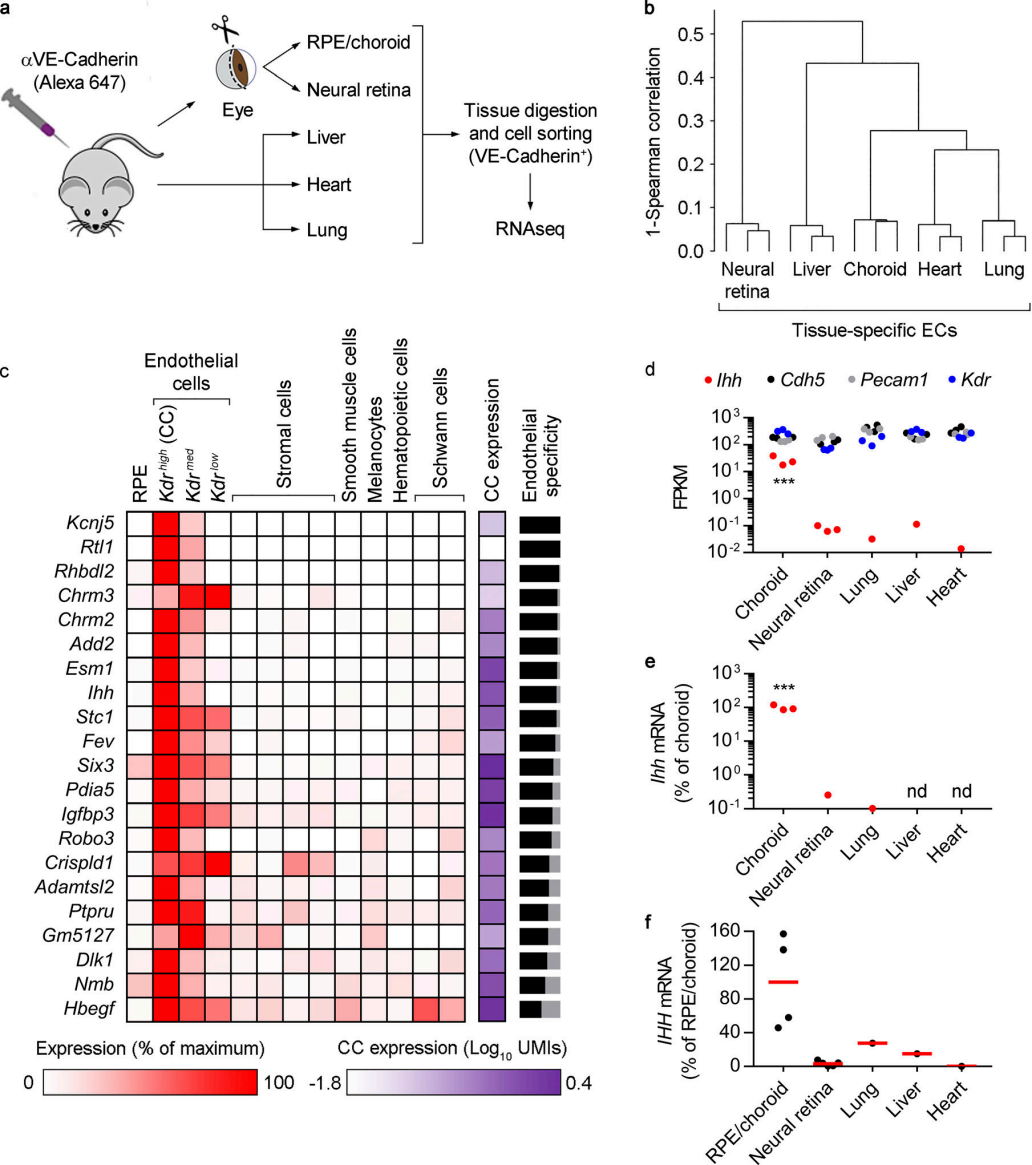
**Transcriptional analysis of tissue-specific mouse ECs and *Ihh* expression in choriocapillaris ECs.** **(a)** Diagram showing the isolation of tissue-specific ECs after intravital EC labeling. For RPE/choroid and neural retina, seven animals (14 eyes) were used per isolation; for lung, liver, and heart, one animal was used per isolation. Bulk RNAseq was performed using RNA from three independent isolations. **(b)** Hierarchical clustering of FPKM profiles showing separate clustering of tissue-specific ECs (*n* = 3). **(c)** Signature genes of mouse choroidal ECs and relative expression among RPE/choroid cell types. Using data from the bulk RNAseq analyses of tissue-specific ECs, the list was assembled by selecting the genes with expression levels at least fivefold higher in choroidal ECs compared with the rest of the tissue-specific ECs (*n* = 3, Benjamini–Hochberg corrected adj P < 0.05), with a detection threshold set at ≥1 FPKM. The list was then interrogated against our scRNAseq data to assess the relative expression of each gene among all cell types, which was calculated using the average normalized UMIs in each cluster and represented as the percentage of the cluster with maximum expression. White, 0%; red, 100%. The column labeled “CC expression” shows the average normalized UMIs (log_10_) for each gene in *Kdr^high^* ECs, i.e., the choriocapillaris (CC), represented in a white-purple scale. Black-gray bars on the right represent EC specificity compared with the rest of RPE/choroid cell types. For each gene, the sum of the average normalized UMIs in *Kdr^high^*, *Kdr^med^*, and *Kdr^low^* ECs was represented as the percentage of total average normalized UMIs (the sum of average normalized UMIs in all clusters). The black portion of the bars represents the percentage of estimated EC specificity. Only genes with >50% EC specificity are shown. **(d)** Bulk RNAseq results showing the expression (FPKM) of *Ihh* (red) and the EC markers *Cdh5* (black), *Pecam1* (gray), and *Kdr* (blue) in tissue-specific ECs (*n* = 3). ***, Benjamini–Hochberg corrected adj P < 0.001 all groups versus choroidal ECs. **(e)** Real-time PCR showing relative *Ihh* expression in tissue-specific ECs. Results are presented as the percentage of the average value for choroidal ECs (*n* = 3, ANOVA + Bonferroni test). ***, P < 0.001 all groups versus choroidal ECs. nd, not detected. **(f)** Real-time PCR showing relative *IHH* expression in human whole tissues (RPE/choroid, *n* = 4; neural retina, *n* = 5; commercially obtained lung, liver, and heart, *n* = 1). Biological replicates are shown as black dots. Red lines show the average expression in RPE/choroid and neural retina. Results are presented as the percentage of the average value for RPE/choroid tissue.

It is becoming evident that adult ECs have tissue-specific transcriptomes reflecting context-dependent physiological and regenerative roles (Rafii et al., 2016). We recently reported the transcriptome of developing and adult mouse choroidal ECs, isolated by intravital staining of the EC marker VE-cadherin, followed by RPE/choroid digestion, flow cytometry sorting, and bulk RNA sequencing (RNAseq; Benedicto et al., 2017). Here, we followed the same approach to compare the transcriptional profile of ECs from adult mouse choroid, neural retina, lung, heart, and liver (Fig. 2 a). Hierarchical clustering analysis demonstrated marked transcriptome differences between the various EC types (Fig. 2 b). We identified a list of 99 “signature genes” of mouse choroidal ECs that were expressed at levels at least fivefold higher than in the other ECs, with a detection threshold set at ≥1 fragments per kilobase per million reads (FPKMs; Data S1 f). By comparing our bulk and single cell RNAseq data, we were able to determine the relative expression of every choroidal EC signature gene relative to other RPE/choroid cell types. Out of 99 genes, 21 were specifically enriched in choroidal ECs (Fig. 2 c; >50% of the sum of average normalized UMIs from all RPE/choroid cell types), from which 16 were significantly more abundant (adj P < 0.05) in *Kdr^high^* (choriocapillaris) ECs than in *Kdr^med^* and *Kdr^low^* choroidal ECs (Data S1, d and g). Our studies provide the first comprehensive list of specific molecular markers for all choroidal ECs relative to not only other choroidal cell types but also other tissue-specific ECs. They demonstrate that the choriocapillaris is the most specialized vascular subtype within the choroid and provide an important platform for future studies on choroid physiopathology and the pathogenesis of choroid-based blinding diseases.

One of the genes specifically enriched in *Kdr^high^* (choriocapillaris) ECs was *Ihh* (Fig. 2 c). The three secreted Hedgehog (HH) proteins (IHH, Sonic Hedgehog, and Desert Hedgehog) act in a paracrine manner, and in most tissues are expressed by epithelial cells and signal to the surrounding mesenchyme (Petrova and Joyner, 2014; Wu et al., 2017). Detailed examination of our bulk RNAseq data revealed that *Ihh* expression in choroidal ECs was >340-fold higher than in the rest of the tissue-specific ECs (Fig. 2 d), an observation that was confirmed by real-time PCR assays (Fig. 2 e). Expression of the generic EC markers *Cdh5*, *Pecam1*, and *Kdr* was similar among all tissue-specific ECs, further highlighting the specificity of *Ihh* enrichment (Fig. 2 d). Importantly, *IHH* expression in human RPE/choroid tissue from healthy donors was markedly higher than in neural retina, lung, liver, and heart, as assessed by real-time PCR (Fig. 2 f). Our observation that *Ihh* is expressed at high levels in adult choriocapillaris is particularly intriguing, as IHH is known to regulate prenatal mouse eye development (Dakubo et al., 2008). However, its expression and potential role in the adult mouse eye has never been studied before. These results prompted us to study the signaling pathway downstream of choroidal EC-expressed *Ihh*.

### Localization and characterization of choroidal GLI1^+^ IHH target cells

**Figure 3.**
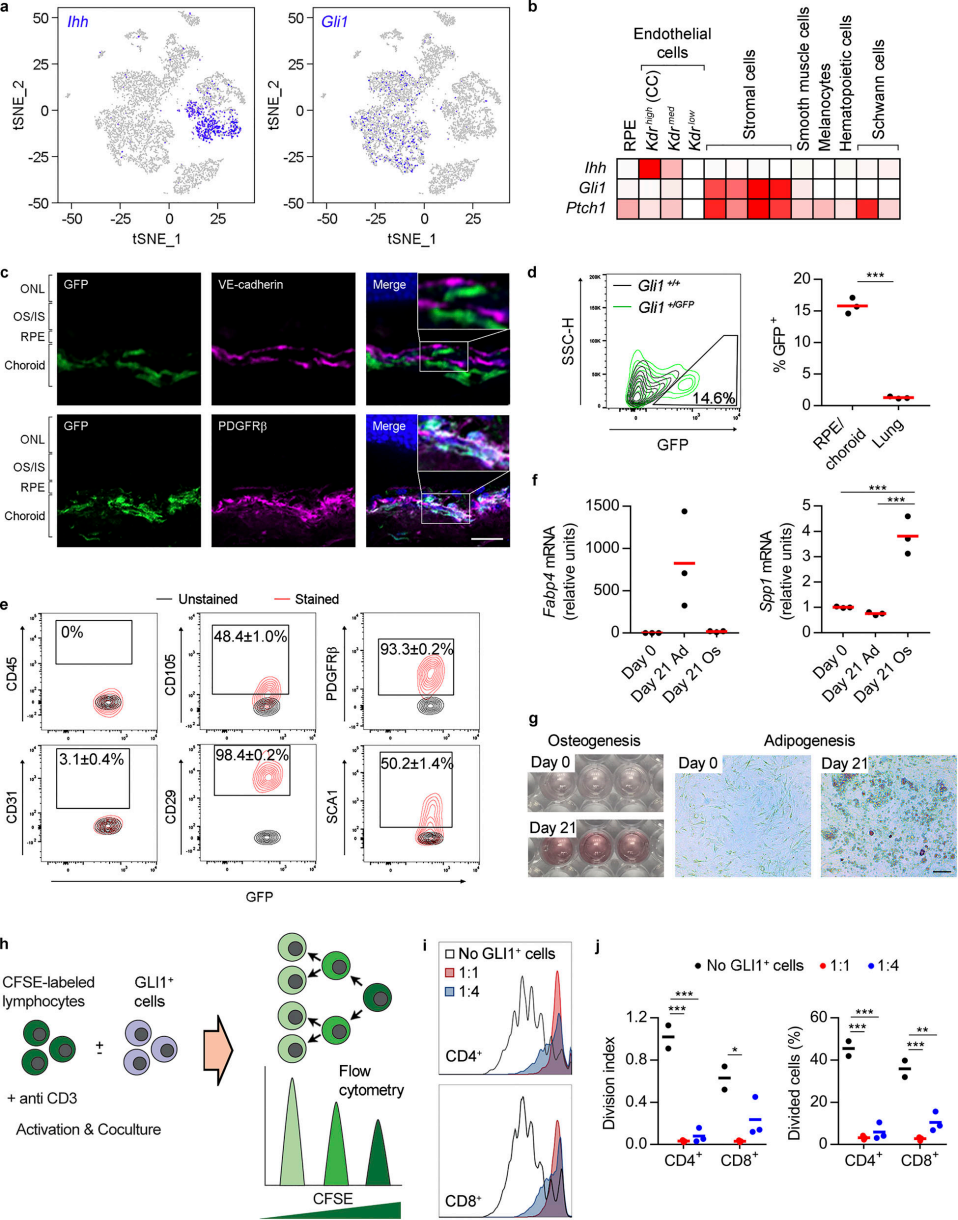
**Characterization of choroidal GLI1^+^ cells.** **(a)** tSNE graphs showing the predominant expression of *Ihh* and *Gli1* in KDR^high^ ECs (CC, choriocapillaris) and stromal cells, respectively. **(b)** Heatmap showing the relative expression of *Ihh*, *Gli1*, and *Ptch1* in RPE/choroid cell types, calculated using the average normalized UMIs in each cluster and represented as the percentage of the cluster with maximum expression. White, 0%; red, 100%. **(c)** Immunofluorescence assays using eye cryosections from *Gli1^GFP/+^* mice show the perivascular localization of choroidal GLI1^+^ cells (GFP^+^, green). Top: Localization of GFP^+^ cells and VE-cadherin^+^ ECs (purple) is mutually exclusive. Bottom: GFP^+^ cells colocalize with the stromal marker PDGFRβ (purple). Colocalization in the merged images is shown in white. Nuclei were stained with Hoechst (blue). Zoomed representative regions of the merged images are shown as insets on the right panels. ONL, outer nuclear layer; OS/IS, outer segments/inner segments. Scale bar, 20 µm. Results are representative of at least two independent experiments. **(d)** Percentage of GLI1^+^ cells (GFP^+^) in RPE/choroid and lung. Left: Representative quantification of choroidal GLI1^+^ cells from *Gli1^GFP/+^* mice by flow cytometry (green lines). *Gli1^+/+^* mice (black lines) were used as a negative control to set background fluorescence levels. Right: Choroid and lung GLI1^+^ cells were quantified by flow cytometry and represented as the percentage of total cells in each tissue (*n* = 3, *t* test). ***, P < 0.001. **(e)** Characterization of choroidal GLI1^+^ cells by flow cytometry. Gated GLI1^+^ cells (GFP^+^) were analyzed for the expression of CD45 (hematopoietic marker), CD31 (EC marker), and the MSC markers CD105, CD29, PDGFRβ, and SCA1. Unstained samples (black lines) were used to set background fluorescence levels. Numbers show the percentage ± SD (*n* = 3) of GFP^+^ cells positive for the different markers. **(f)** Choroidal GLI1^+^ cells were isolated from *Gli1^GFP/+^* mice by cell sorting (GFP^+^ cells) and expanded in culture. GLI1^+^ cells were exposed to adipogenic (Ad) or osteogenic (Os) media, and expression of *Fabp4* and *Spp1* (adipocyte and osteoblast markers, respectively) was assessed by real-time PCR at days 0 and 21 of differentiation (*n* = 3, ANOVA + Bonferroni test). ***, P < 0.001. **(g)** GLI1^+^ cells were cultured in osteogenic (left) or adipogenic (right) media for 21 d. Osteogenic and adipogenic differentiation was assessed by the appearance of calcium deposits (Alizarin red S staining) or intracellular lipid vesicles (oil red O staining), respectively. Day 0 cultures were included as controls. **(h)** Inhibition of T cell proliferation by GLI1^+^ cells (experiment outline). CFSE-labeled allogeneic mouse splenocytes were activated with anti-CD3 antibody and cultured alone or in the presence of choroid GLI1^+^ cells at different ratios (GLI1^+^ cells/splenocytes, 1:1 and 1:4) for 4 d. **(i)** CFSE dilution histograms showing that the proliferation of CD4^+^ and CD8^+^ T cells (white histogram) is strongly inhibited by choroidal GLI1^+^ cells at both 1:1 and 1:4 ratios. **(j)** Quantification of panel i using the division index (average number of divisions after stimulation) and the percentage of divided cells (No GLI1^+^ cells, *n* = 2; 1:1, *n* = 3; 1:4, *n* = 3; ANOVA + Bonferroni test). *, P < 0.05; **, P < 0.01; ***, P < 0.001. Results in panels f–j are representative of two independent experiments.

Because IHH is a secreted protein expected to signal to the surrounding mesenchymal cells, we sought to identify cell types within RPE/choroid capable of responding to EC-expressed *Ihh*. Visualization of *Ihh*-expressing cells on t-distributed stochastic neighbor embedding (tSNE) plots confirmed that *Ihh* transcripts are constitutively enriched in the *Kdr*^*high*^ subcluster of ECs, while the expression of the gene encoding the transcriptional activator GLI1, a transcriptional target dependent on HH signaling and thus a readout of canonical HH pathway activity (Ahn and Joyner, 2005; Bai et al., 2004; Petrova and Joyner, 2014; Wu et al., 2017), was specifically increased in stromal cell clusters 5–8 relative to other choroidal cell types (Fig. 3 a). More detailed analysis of our scRNAseq data confirmed the stromal specificity of *Gli1* expression (Fig. 3 b). Although the *Ptch1* gene encoding the HH receptor presented a broader expression pattern than *Gli1*, it was clearly enriched in stromal cells (Fig. 3 b). This is consistent with the fact that *Ptch1*, in addition to encoding the HH receptor, is up-regulated in response to HH signaling (Petrova and Joyner, 2014; Wu et al., 2017). To spatially localize choroidal GLI1^+^ cells, we used albino *Gli1^GFP/+^* reporter mice in which GFP expression is controlled by the endogenous *Gli1* promoter of one of the alleles (Brownell et al., 2011). Thus, HH-responding cells can be readily identified as GFP^+^ cells. Immunostaining analysis of chorioretinal sections from *Gli1^GFP/+^* mice showed that GFP^+^ cells were primarily localized surrounding VE-cadherin^+^ ECs and colocalized with the stromal marker PDGFRβ (Fig. 3 c). These results indicate that the main paracrine targets of EC-secreted IHH are perivascular stromal cells.

**Figure S2.**
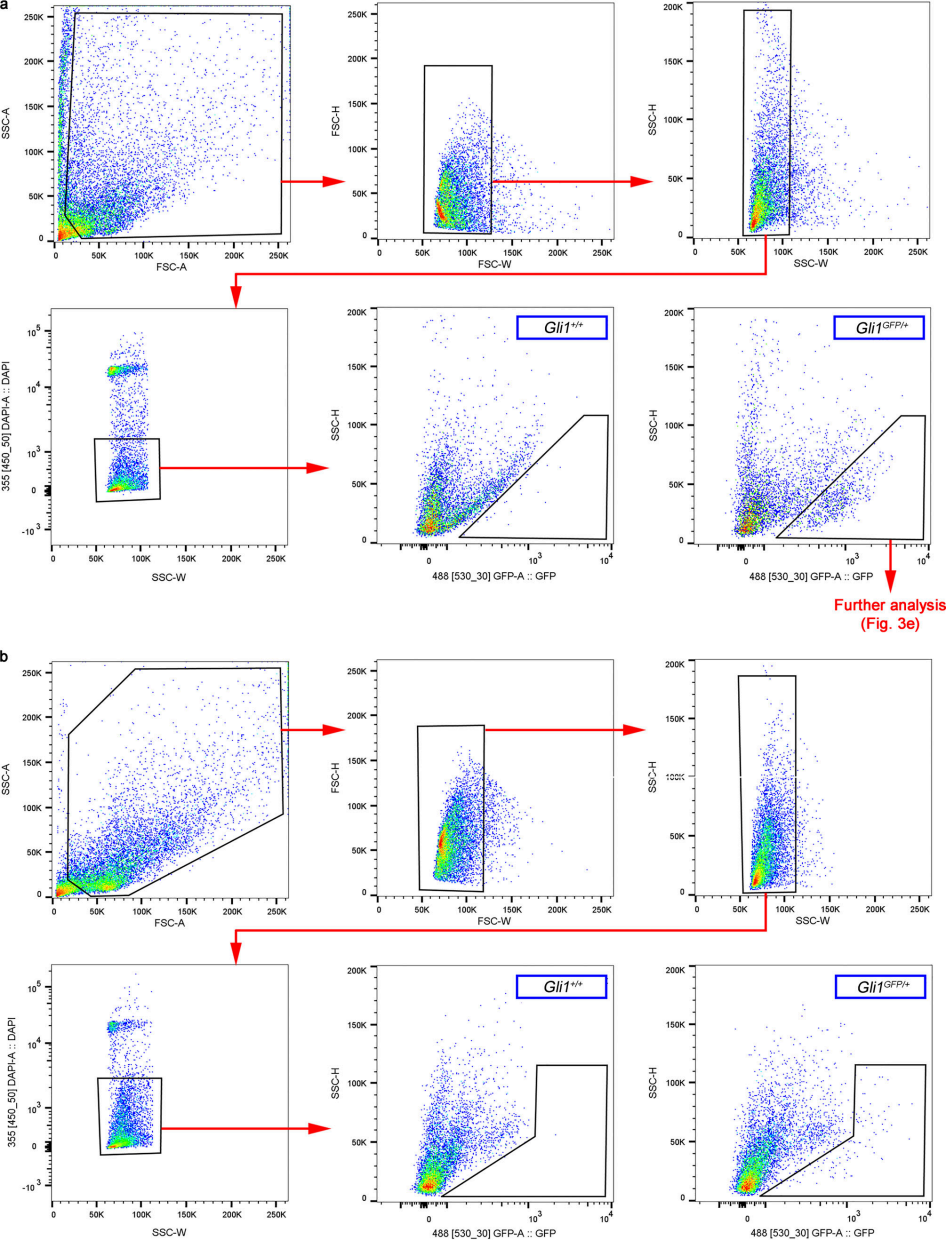
**Gating strategy to quantify and characterize GLI1^+^ cells from *Gli1^GFP/+^* mice by flow cytometry.** **(a and b)** Analysis of GFP^+^ cells from RPE/choroid (a) and lung (b). Tissue from *Gli1^+/+^* mice was used as negative control. FSC-H, forward scatter height; SSC-A, side scatter area; SSC-H, side scatter height; SSC-W, side scatter width.

Previous work demonstrated the constitutive presence of perivascular GLI1*^+^* MSC-like cells in several adult mouse tissues. These cells represent between 0.01% and 0.17% of total tissue cells and are capable of differentiating into adipocytes, osteoblasts, and chondrocytes in vitro (Kramann et al., 2015). To assess their potential similarity with choroidal GLI1*^+^* cells described here, we first studied the percentage of GFP^+^ cells in whole digested tissue by flow cytometry. We found that the population of resident GFP^+^ cells in RPE/choroid was 15.8 ± 1.3%, a much higher percentage compared with lung tissue (1.28 ± 0.2%; Fig. 3 d and Fig. S2). Next, we phenotyped choroidal GFP^+^ cells by flow cytometry studying the expression of a repertoire of cell surface markers previously used to characterize GLI1*^+^* MSC-like cells in other tissues (Kramann et al., 2015). We found that virtually all choroidal GFP^+^ cells were positive for PDGFRβ and CD29 and negative for the hematopoietic lineage marker CD45 and the EC marker CD31, and ∼50% expressed CD105 and SCA1 (Fig. 3 e). To study whether choroidal GLI1*^+^* cells are pluripotent in vitro, we isolated GLI1*^+^* cells from RPE/choroid of *Gli1^GFP/+^* mice by cell sorting based on GFP expression. Isolated GFP^+^ cells were plastic adherent and could be differentiated into osteoblast- and adipocyte-like cells (Fig. 3, f and g). Since it is well established that MSCs inhibit T cell proliferation in vitro (Uccelli et al., 2008), we tested whether choroidal GLI1^+^ cells exert the same effect. Mouse splenocytes were labeled with CFSE, polyclonally activated, and cultured in the absence or presence of allogeneic choroidal GLI1^+^ cells. Proliferation of CD4^+^ and CD8^+^ T cells was inhibited in the presence of GLI1^+^ cells in a dose-dependent manner (Fig. 3, h–j). Overall, our characterization of choroidal GLI1*^+^* cells is in agreement with the MSC-like identity previously reported for GLI1*^+^* perivascular cells from other tissues (Kramann et al., 2015).

### RPE/choroid transcriptional alterations after EC-specific deletion of *Ihh* in adult mice

**Figure 4.**
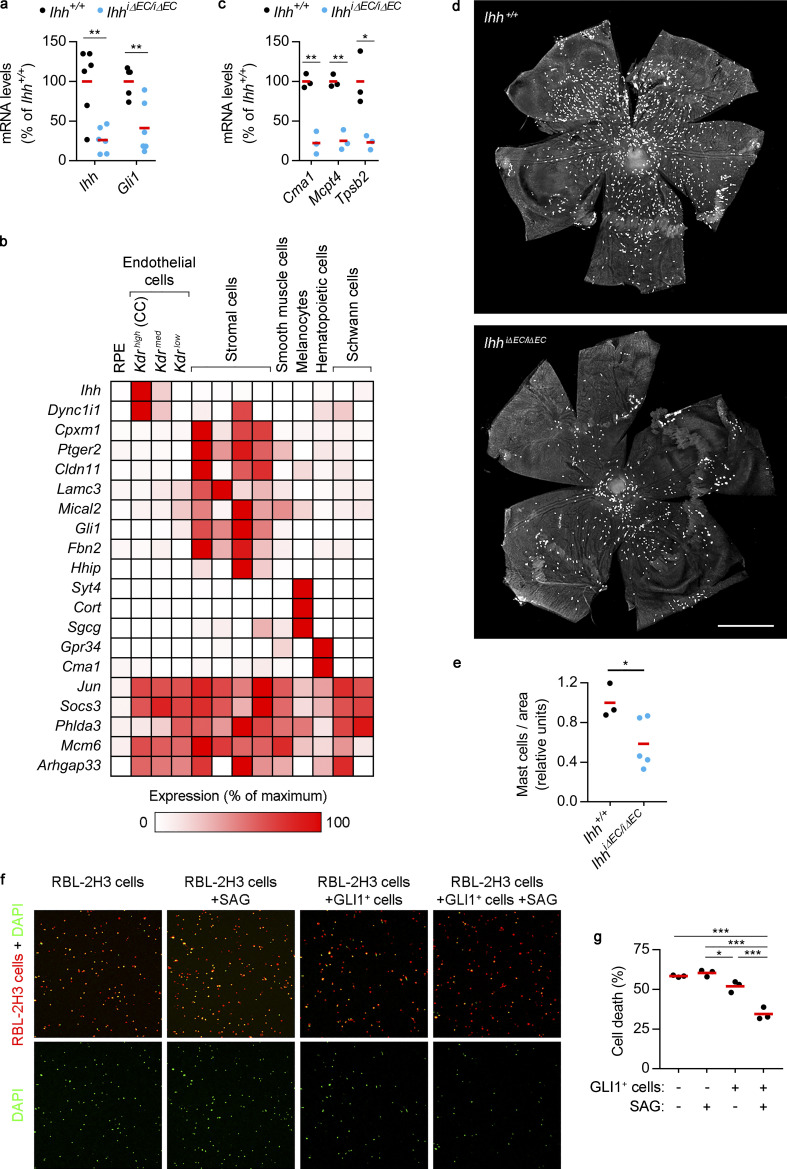
**RPE/choroid transcriptional alterations and loss of choroidal mast cells after EC-specific *Ihh* deletion.** **(a)** Real-time PCR showing *Ihh* and *Gli1* expression levels in RPE/choroid tissue from *Ihh^+/+^* and *Ihh^iΔEC/iΔEC^* mice (black and blue dots, respectively). Individual dots correspond to different animals, and red bars represent average values. Relative gene expression is presented as the percentage of *Ihh^+/+^* mice (*n* = 6, *t* test). **, P < 0.01. **(b)** RNAseq analysis of RPE/choroid tissue from *Ihh^+/+^* and *Ihh^iΔEC/iΔEC^* mice and cross-comparison with scRNAseq data. The heatmap shows genes with ≥1 FPKM in *Ihh^+/+^* mice that were significantly down-regulated in *Ihh^iΔEC/iΔEC^* mice (*n* = 3, at least twofold change, Benjamini–Hochberg corrected adj P < 0.05). Their relative expression across RPE/choroid cell types was calculated using the average normalized UMIs in each cluster and represented as the percentage of the cluster with maximum expression. White, 0%; red, 100%. **(c)** Real-time PCR showing the expression of mast cell markers in RPE/choroid tissue from *Ihh^+/+^* and *Ihh^iΔEC/iΔEC^* mice (*n* = 3, *t* test). *, P < 0.05; **, P < 0.01. Results are represented as in panel a. **(d)** Representative avidin staining assay to localize choroidal mast cells using flatmounts from albino *Ihh^+/+^* and *Ihh^iΔEC/iΔEC^* mice. Scale bar, 1,000 µm. **(e)** Quantification of avidin staining assays shown in panel d. Results are expressed as number of mast cells normalized by flatmount area and presented as relative units (*Ihh^+/+^*, *n* = 3; *Ihh^iΔEC/iΔEC^,* *n* = 5, *t* test). *, P < 0.05. **(f)** In vitro assays to study the survival of serum-starved RBL-2H3 cells (red) in the absence or presence of choroidal GLI1^+^ cells and the HH pathway agonist SAG. Dead cells are DAPI^+^ (green). **(g)** Quantification of RBL-2H3 cell death shown in panel f. The graph shows the percentage of DAPI^+^ RBL-2H3 cells. Black dots correspond to biological replicates, and red bars represent average values (*n* = 3, ANOVA + Bonferroni test). Results are representative of two independent experiments. *, P < 0.05; ***, P < 0.001.

To directly study the role of choroidal EC-expressed *Ihh* in choroidal and retinal homeostasis of adult mice, we generated a mouse model that enabled EC-specific, inducible *Ihh* deletion. *Cdh5*-PAC-Cre^ERT2^ mice, in which a tamoxifen-inducible Cre is expressed under the control of the EC-specific *Cdh5* promoter (Wang et al., 2010), were crossed with mice harboring floxed *Ihh* to obtain *Cdh5*-PAC-Cre^ERT2^ *Ihh^loxP/loxP^* mice (hereafter termed *Ihh^iΔEC/iΔEC^*). To assess *Ihh* deletion efficiency in our inducible system, we treated control (*Cdh5*-PAC-Cre^ERT2^ *Ihh^+/+^*, hereafter termed *Ihh^+/+^*) and *Ihh^iΔEC/ΔiEC^* adult mice with tamoxifen and assessed *Ihh* mRNA levels in RPE/choroid by real-time PCR. We observed that *Ihh^iΔEC/iΔEC^* mice presented a ∼70% reduction in *Ihh* mRNA levels. Confirming that IHH signaling is diminished in mutants, *Gli1* expression was similarly decreased (Fig. 4 a). These results confirmed our scRNAseq data showing that the main source of *Ihh* expression in RPE/choroid tissue are choroidal ECs and, importantly, demonstrate that choroidal *Gli1* expression depends on EC-expressed *Ihh*.

Next, we performed bulk RNAseq analyses of RPE/choroid tissue obtained from tamoxifen-treated *Ihh^+/+^* and *Ihh^iΔEC/iΔEC^* mice (Data S1 h). To analyze the impact of EC-specific *Ihh* deletion on the different cell types within RPE/choroid tissue, we assembled a list of genes whose expression in *Ihh^+/+^* mice was ≥1 FPKM and at least twofold higher than in *Ihh^iΔEC/iΔEC^* mice (adj P < 0.05). In other words, we selected for genes highly expressed in RPE/choroid that were markedly down-regulated after EC-specific *Ihh* deletion. We then used our scRNAseq analyses of wild-type mice to plot the relative expression of each of these genes in all RPE/choroid cell clusters (Fig. 4 b). We found that relative expression of these genes in RPE was very low. This result indicated that, at least under basal conditions, EC-specific *Ihh* deletion had little impact on RPE transcriptome compared with the rest of the cell types. Out of the 20 genes included in the list, we found 8 that were clearly enriched in stromal cell clusters 5–8, including the HH-induced genes *Gli1* and *Hhip*. This finding was expected, given our previous observation that stromal cells are the main choroidal *Ihh* target. Interestingly, EC-specific *Ihh* deletion not only altered genes expressed by HH-responding stromal cells but also down-regulated genes expressed almost exclusively in other choroidal cell populations that are not direct targets of IHH. That was the case for *Syt4*, *Cort*, and *Sgcg*, which are highly enriched in melanocytes, and *Gpr34* and *Cma1*, which are selectively detected in hematopoietic cells. These results strongly suggest that deletion of EC-expressed *Ihh* indirectly affects choroidal melanocytes and hematopoietic cells. Finally, a group of genes (*Jun*, *Socs3*, *Phlda3*, *Mcm6*, and *Arhgap33*) presented homogeneous relative expression in many choroidal cell types. However, because clusters 5–8 constitute ∼50% of the total cellular content of RPE/choroid tissue (Table 1), this analysis strategy is not appropriate to elucidate whether the decreased expression of these genes by at least twofold in the RPE/choroid of *Ihh^iΔEC/iΔEC^* mice results from their down-regulation in choroidal stromal cells, nonstromal cells, or both. Taken together, our bioinformatics analyses suggest that EC-expressed *Ihh* directly regulates gene expression in GLI1*^+^* perivascular MSC-like cells, which may in turn induce alterations in other choroidal cell types, including melanocytes and hematopoietic cells.

### Impairment of HH signaling causes loss of choroidal mast cells

**Figure S3.**
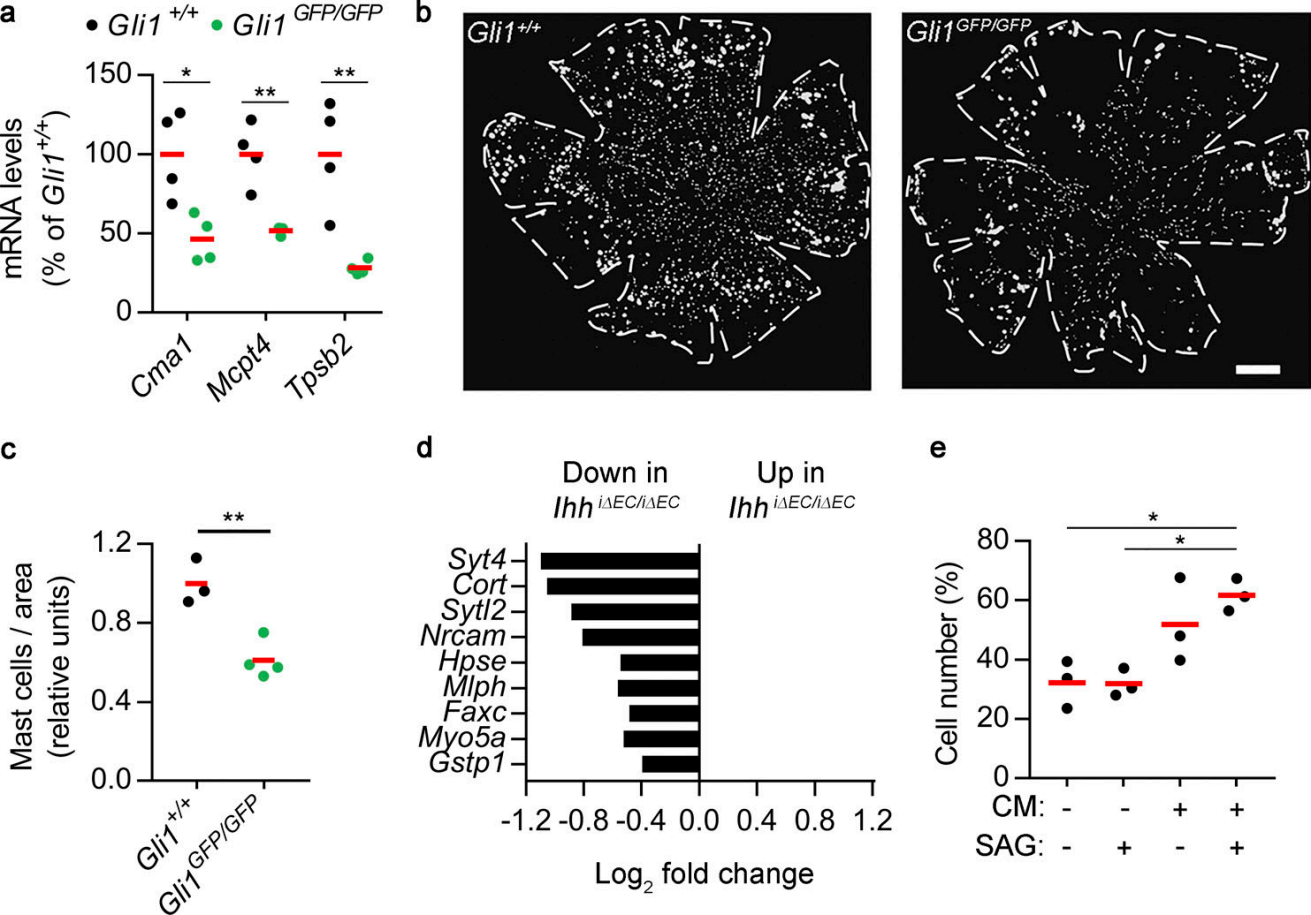
**Effects of HH signaling on choroidal mast cells and melanocyte proliferation.** **(a)** Real-time PCR showing the expression of mast cell markers in RPE/choroid tissue from *Gli1^+/+^* and *Gli1^GFP/GFP^* mice (black and green dots, respectively). Individual dots correspond to different animals, and red bars represent average values. Relative gene expression is presented as the percentage of *Gli1^+/+^* mice (*n* = 4, *t* test). *, P < 0.05; **, P < 0.01. **(b)** Representative avidin staining assay to localize choroidal mast cells using flatmounts from albino *Gli1^+/+^* and *Gli1^GFP/GFP^* mice. Panels show binary images used for segmentation, and dashed lines delineate flatmount contours. Scale bar, 500 µm. **(c)** Quantification of avidin staining assays shown in panel b. Results are expressed as number of mast cells normalized by flatmount area and presented as relative units (*Gli1^+/+^*, *n* = 3; *Gli1^GFP/GFP^*, *n* = 4; *t* test). **, P < 0.01. **(d)** Differential expression of melanocyte-enriched genes in RPE/choroid from *Ihh^+/+^* and *Ihh^iΔEC/iΔEC^* mice as determined by bulk RNAseq (*n* = 3, Benjamini–Hochberg corrected adj P < 0.05). **(e)** Serum-starved melan-a cells were grown in culture medium alone, culture medium supplemented with 100 nM SAG, or conditioned media (CM) from unstimulated or 100 nM SAG–treated GLI1^+^ cells. All conditions included 0.5% FBS. After 48 h, cell number was assessed and represented as the percentage of cells grown in normal melan-a culture medium with 10% FBS. Individual dots correspond to three independent experiments performed in biological triplicates, and red bars represent average values (*n* = 3, ANOVA + Bonferroni test). *, P < 0.05.

*Cma1* encodes chymase 1, a protease specifically expressed by mast cells (Dwyer et al., 2016). Mast cells control innate and adaptive immune responses and trigger IgE-associated allergic inflammation (Galli et al., 2008). Although the existence of choroidal mast cells in humans and rodents is well established (May, 1999; McMenamin, 1997; McMenamin and Polla, 2013), their role in the choroid is unknown. The decrease of *Cma1* expression in *Ihh^iΔEC/iΔEC^* mice (Fig. 4 b) suggested that EC-specific *Ihh* expression may control the number and function of choroidal mast cells. We reanalyzed our RNAseq data in search for other mast cell specific markers (Dwyer et al., 2016) and found that in addition to *Cma1* (adj P = 0.02), *Mcpt4* and *Tpsb2* expression was also reduced in *Ihh^iΔEC/iΔEC^* mice more than twofold (adj P = 0.07 and 0.06, respectively; Data S1 h), albeit not reaching the statistical significance threshold of adj P < 0.05 used to filter data shown in Fig. 4 b. Real-time PCR assays revealed significantly decreased expression of *Cma1*, *Mcpt4*, and *Tpsb2* in RPE/choroid tissue from *Ihh^iΔEC/iΔEC^* mice (Fig. 4 c), suggesting that EC-specific *Ihh* deletion may result in reduced numbers of choroidal mast cells. To address this possibility, we performed avidin staining assays to detect mast cells (Tharp et al., 1985; Veerappan et al., 2008) in RPE/choroid flatmounts. Because RPE and choroidal pigmentation interferes with flatmount imaging, these experiments were performed using albino mice generated by breeding the C57BL/6J-derived *Ihh^iΔEC/iΔEC^* pigmented line with albino mice. Quantitative image analysis showed reduced numbers of choroidal mast cells in albino mice after EC-specific *Ihh* deletion (Fig. 4, d and e). Next, we tested whether alteration of the HH pathway in GLI1^+^ cells also resulted in reduced numbers of choroidal mast cells. We intercrossed albino *Gli1^GFP/+^* animals to obtain *Gli1^GFP/GFP^* mice, which are indeed null for *Gli1* function. Although *Gli1* is dispensable for normal mouse development (Bai et al., 2002; Park et al., 2000), *Gli1* deletion induces altered phenotypes in particular contexts (Lees et al., 2008; Merchant et al., 2010). Real-time PCR assays showed that expression of *Cma1*, *Mcpt4*, and *Tpsb2* in RPE/choroid tissue from *Gli1^GFP/GFP^* mice was significantly reduced compared with *Gli1^+/+^* mice (Fig. S3 a). Avidin staining assays showed reduced numbers of choroidal mast cells in *Gli1^GFP/GFP^* mice (Fig. S3, b and c), confirming the importance of HH signaling for choroidal mast cell fate. These results demonstrate that blunting HH signaling, either by decreasing ligand levels (IHH) or impairing target cell response (*Gli1* deletion), results in reduced numbers of choroidal mast cells. One attractive explanation for this observation is that mast cell survival in the choroid may rely on factors provided by HH-stimulated GLI1^+^ cells. As a proxy to test this hypothesis, we performed in vitro assays using RBL-2H3 cells, a cell line widely used to study some features of mast cell physiology (Passante and Frankish, 2009). Fluorescently labeled RBL-2H3 cells were cultured alone or together with GLI1^+^ cells, in the absence or presence of the HH pathway agonist Smoothened agonist (SAG; Chen et al., 2002). After 24 h of serum starvation, viability of nonadherent RBL-2H3 cells was evaluated by DAPI staining. The presence of GLI1^+^ cells reduced the percentage of dead RBL-2H3 cells (DAPI^+^), which was further decreased by the addition of SAG (Fig. 4, f and g). Importantly, SAG treatment in the absence of GLI1^+^ cells had no effect on RBL-2H3 cell viability. In sum, our in vivo and in vitro assays support a model in which EC-secreted IHH stimulates choroidal GLI1^+^ cells to promote mast cell survival.

### HH-stimulated GLI1^+^ cells induce melanocyte proliferation

Because reduced RPE/choroid expression of mast cell markers correlated well with the actual number of choroidal mast cells, we reasoned that reduced expression of the melanocyte-enriched genes *Syt4*, *Cort*, and *Sgcg* in the RPE/choroid of *Ihh^iΔEC/iΔEC^* mice (Fig. 4 b) may also indicate loss of choroidal melanocytes. We used our scRNAseq data (Data S1 c) to compile a complete list of choroidal melanocyte-enriched genes (average normalized UMIs at least fivefold higher compared with other cell types; adj P < 0.05) and analyzed their expression in the RPE/choroid of *Ihh^+/+^* and *Ihh^iΔEC/iΔEC^* mice as determined by bulk RNAseq (Data S1 h). We found a trend of reduced expression in *Ihh^iΔEC/iΔEC^* mice (65 out of 70 melanocyte-enriched genes; Data S1 i), and all genes with significant differential expression (adj P < 0.05) were down-regulated in *Ihh^iΔEC/iΔEC^* mice (Fig. S3 d). Conversely, using the same approach, we observed that *Ihh^+/+^* and *Ihh^iΔEC/iΔEC^* mice expressed similar levels of all smooth muscle cell– and choriocapillaris-enriched genes (with the exception of *Ihh* for the latter, as expected), suggesting that the reduction of melanocyte markers was specific (Data S1 i). These observations indicate that EC-specific *Ihh* deletion results in reduced numbers of choroidal melanocytes. To directly test whether HH signaling modulates melanocyte proliferation, we performed in vitro experiments using choroidal GLI1^+^ cells and the mouse melanocyte cell line melan-a (Bennett et al., 1987). Conditioned media from SAG-stimulated GLI1^+^ cells, but not SAG alone, significantly increased the proliferation of serum-starved melanocytes (Fig. S3 e). These results indicate that, in addition to promoting mast cell survival, HH-stimulated choroidal GLI1^+^ cells may modulate the homeostasis of choroidal melanocytes.

### HH signaling modulates the expression of inflammation-related genes in choroidal GLI1^+^ cells

**Figure 5.**
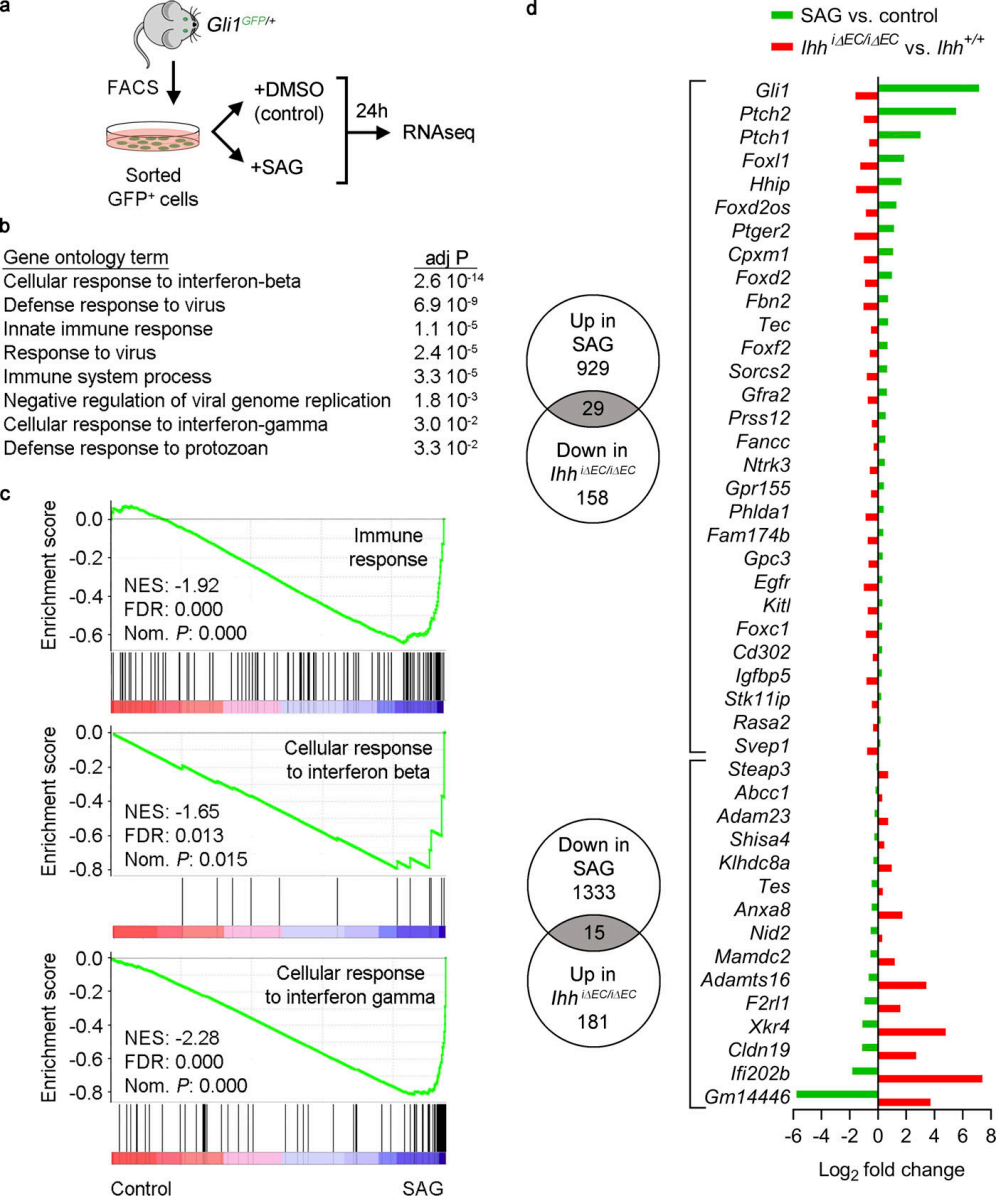
**HH signaling modulates the expression of inflammation-related genes in choroidal GLI1^+^ cells.** **(a)** Experiment outline. GLI1^+^ cells (GFP^+^) were isolated from RPE/choroid tissue of *Gli1^GFP/+^* mice by FACS. Cultured cells were exposed to the HH pathway agonist SAG (100 nM) or vehicle (DMSO) for 24 h, and gene expression was analyzed by RNAseq. **(b)** Biological process (GOTERM_BP_DIRECT) Gene Ontology analysis of control- and SAG-treated GLI1^+^ cells using DAVID software, considering genes with log_2_ fold change ≤−1 and ≥1 (*n* = 3, Benjamini–Hochberg corrected adj P < 0.05). Significantly enriched gene sets are shown (Benjamini-corrected adj P < 0.05). **(c)** Pre-ranked GSEA shows that SAG treatment reduces the expression of immune-related genes. FDR, false discovery rate q-value; NES, normalized enrichment score; Nom. *P*, nominal P value. **(d)** Genes with inversely correlated differential expression between HH activation in vitro (GLI1^+^ cells after SAG treatment) and inhibition in vivo (RPE/choroid tissue after EC-specific *Ihh* deletion). Numbers in Venn diagrams indicate the number of genes significantly up- or down-regulated in each condition (Benjamini–Hochberg corrected adj P < 0.05). Gray intersections indicate the number of overlapping genes for each comparison, which are listed on the graph on the right. Green bars represent genes up- or down-regulated in SAG-treated GLI1^+^ cells compared with control cells, and red bars represent genes up- or down-regulated in RPE/choroid tissue from *Ihh^iΔEC/iΔEC^* mice compared with *Ihh^+/+^* mice.

To gain further insight into the crosstalk mechanisms and functional roles of the HH pathway in the choroid, we performed RNAseq analyses of cultured choroidal GLI1^+^ cells in the absence or presence of the HH pathway agonist SAG (Fig. 5 a and Data S1 j). Gene Ontology analyses of up- and down-regulated genes (at least twofold) using DAVID software (Huang et al., 2009) showed that the most significant transcriptome changes upon HH pathway activation corresponded to genes related to immune response, including the categories cellular response to IFN-β, cellular response to IFN-γ, and innate immune response (Fig. 5 b). Gene set enrichment analysis (GSEA; Subramanian et al., 2005) confirmed that SAG treatment significantly reduced the expression of genes related to immune response and cellular response to IFN-β and IFN-γ (Fig. 5 c). Moreover, Ingenuity Pathway Analysis (Qiagen) showed that HH pathway activation significantly inhibited transcriptional networks downstream IFN-β, IFN-γ, several IFN regulatory factors, and proinflammatory receptors and transcriptional regulators such as IL-6R, IL-1R1, STAT1, and NF-κB (Data S1 k). Conversely, HH activation resulted in gene expression changes that mimicked transcriptional events downstream of IL-10RA, the receptor for the anti-inflammatory cytokine IL-10 (Data S1 k). Next, we sought to assess the contribution of such crosstalk mechanism to the transcriptional changes induced in RPE/choroid tissue after EC-specific *Ihh* deletion. Using our RNAseq data, we selected genes whose fold change expression in SAG-treated GLI1^+^ cells (adj P < 0.05 versus control cells) was inversely correlated to the fold change observed in RPE/choroid from *Ihh^iΔEC/iΔEC^* mice (adj P < 0.05 versus *Ihh^+/+^* mice; Fig. 5 d). Out of 158 genes significantly down-regulated in *Ihh^iΔEC/iΔEC^* mice, 29 were up-regulated in SAG-treated GLI1^+^ cells. As expected, these genes included HH downstream targets such as *Gli1*, *Ptch1*, *Foxl1*, and *Hhip*. On the other hand, RPE/choroid from *Ihh^iΔEC/iΔEC^* mice presented increased expression of 181 genes, 15 of which were down-regulated in SAG-treated GLI1^+^ cells. Interestingly, genes with the highest differential expression of this list included the IFN-activated genes *Ifi202b* and *Gm14446* (*Ifit1bl1*), as well as *F2rl1*, which encodes the proinflammatory receptor PAR2 (Déry et al., 1998; Steinhoff et al., 2000). Collectively, our bioinformatics analyses strongly suggest that HH signaling blunts the transcriptional changes of choroidal GLI1*^+^* cells in response to proinflammatory factors. Thus, crosstalk between EC-secreted IHH and stromal GLI1^+^ cells may be key for the maintenance of choroidal immune homeostasis.

### EC-specific *Ihh* deletion alters choroidal and retinal inflammatory responses and aggravates visual function impairment after tissue damage

**Figure 6.**
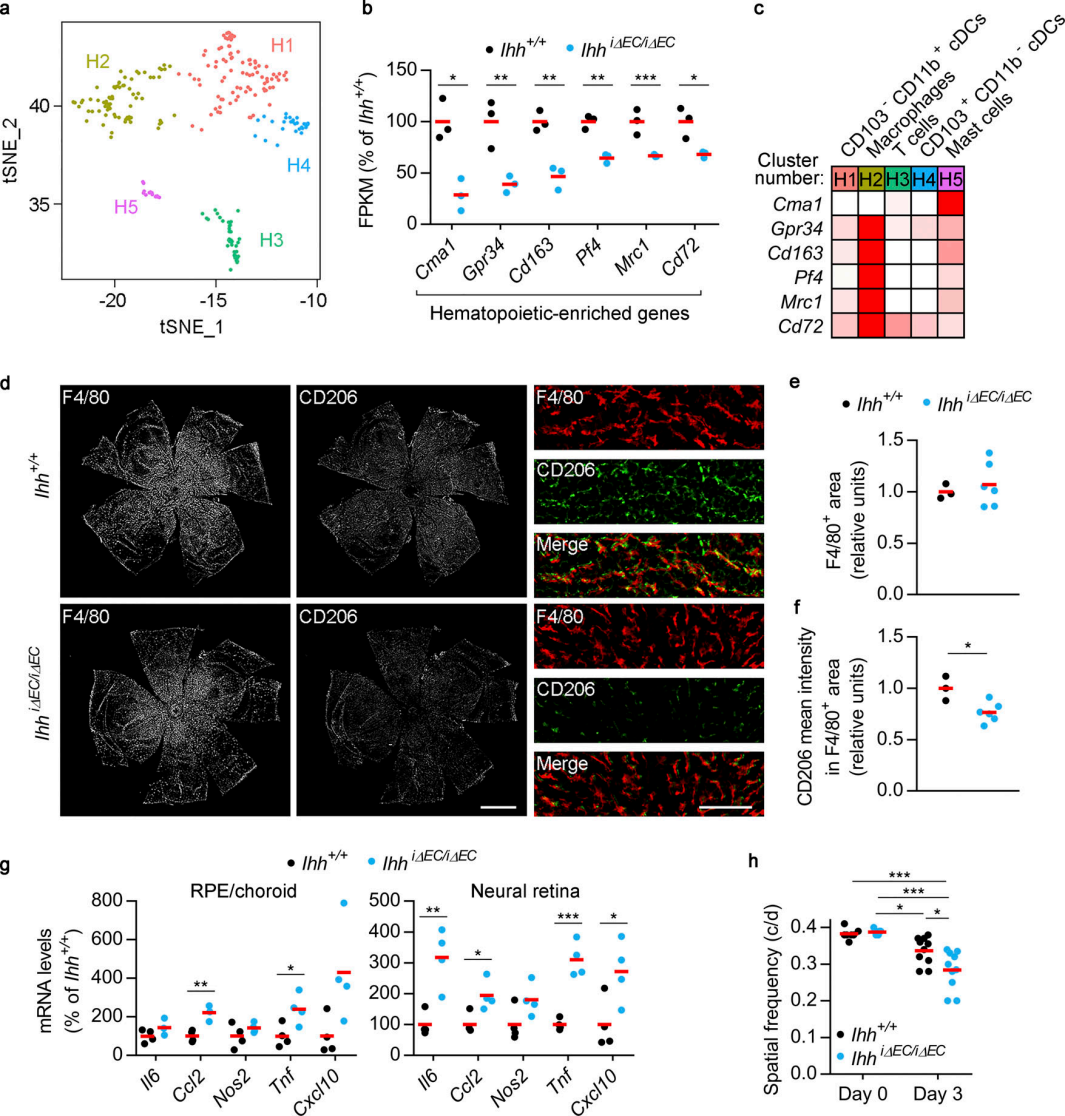
**EC-specific *Ihh* deletion alters choroidal and retinal inflammatory responses and aggravates visual function impairment after tissue damage.** **(a)** tSNE graph showing five transcriptionally distinct subclusters (H1–H5) within choroidal hematopoietic cells, as determined by scRNAseq. **(b)** RNAseq data showing genes significantly enriched in choroidal hematopoietic cells (as determined by our scRNAseq results) and down-regulated in RPE/choroid from *Ihh^iΔEC/iΔEC^* mice (blue dots) compared with *Ihh^+/+^* mice (black dots). Individual dots correspond to different animals, and red bars represent average values. Relative gene expression is presented as the percentage of *Ihh^+/+^* mice (*n* = 3, Benjamini–Hochberg corrected adj P value). *, P < 0.05; **, P < 0.01; ***, P < 0.001. **(c)** Relative expression levels of the genes shown in panel b among choroidal hematopoietic subclusters calculated using the average normalized UMIs in each subcluster and represented as the percentage of the subcluster with maximum expression. White, 0%; red, 100%. **(d)** Immunofluorescence analysis of F4/80 and CD206 expression in RPE/choroid flatmounts from albino *Ihh^+/+^* and *Ihh^iΔEC/iΔEC^* mice. Scale bar, 1,000 µm. Panels on the right show zoomed-in regions (scale bar, 125 µm). **(e)** Quantification of F4/80^+^ area shown in panel d. Results are normalized by flatmount area and presented as relative units (*Ihh^+/+^*, *n* = 3; *Ihh^iΔEC/iΔEC^,* *n* = 6, *t* test). **(f)** Quantification of CD206 intensity within F4/80^+^ area shown in panel d. Results are presented as relative units (*Ihh^+/+^*, *n* = 3; *Ihh^iΔEC/iΔEC^,* *n* = 6, *t* test). *, P < 0.05. **(g)** Real-time PCR assays showing relative expression levels of pro-inflammatory genes in RPE/choroid (left) and neural retina (right) from *Ihh^+/+^* and *Ihh^iΔEC/iΔEC^* mice 3 d after i.v. administration of 15 mg kg^−1^ NaIO_3_. Results are presented as in panel a (*n* = 4, *t* test). *, P < 0.05; **, P < 0.01; ***, P < 0.001. **(h)** Optomotor response analyses of *Ihh^+/+^* and *Ihh^iΔEC/iΔEC^* mice before (day 0) and after (day 3) NaIO3 administration (day 0, *n* = 7; day 3, *n* = 10; ANOVA + Bonferroni test). *, P < 0.05; ***, P < 0.001. c/d, cycles per degree.

**Figure S4.**
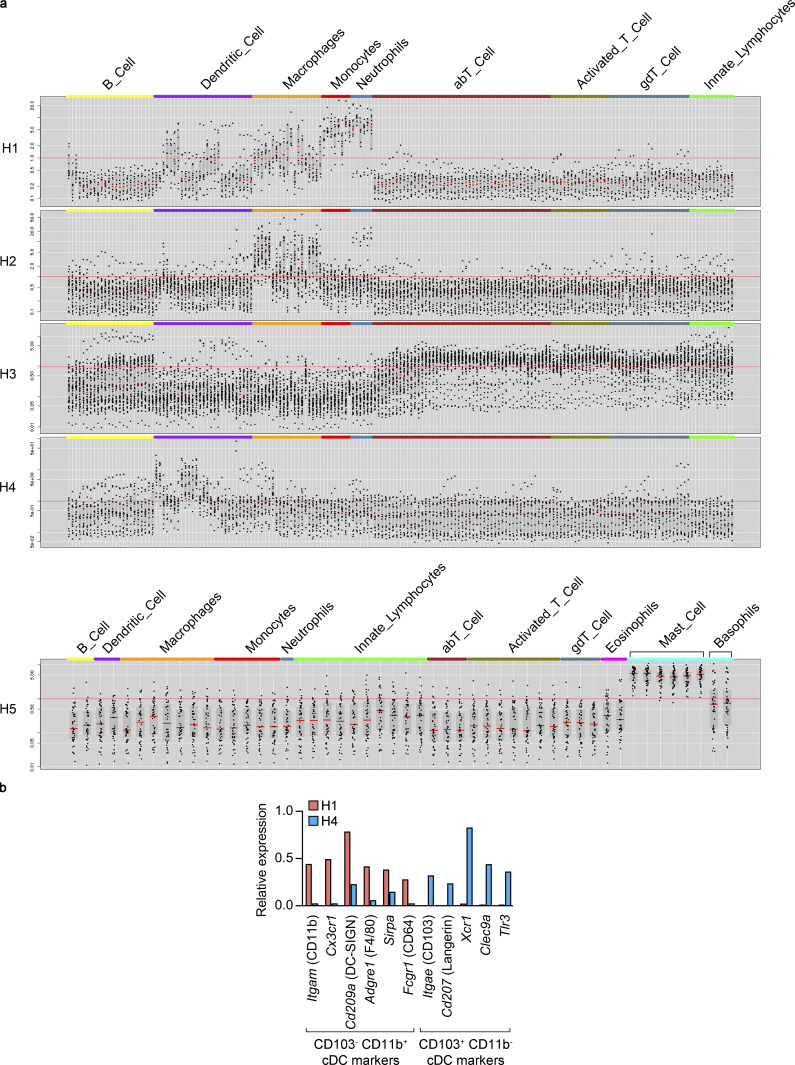
**Classification of choroidal hematopoietic cell subclusters.** **(a)** Gene lists from Data S1 m were analyzed at the Immunological Genome Project website (http://www.immgen.org) using My GeneSet function (V1 datasets for subclusters H1-4, V2 datasets for subcluster H5). **(b)** Expression of CD103^−^ CD11b^+^ and CD103^+^ CD11b^−^ cDC markers (based on Merad et al., 2013) by H1 and H4 subclusters. Average normalized UMIs for each gene and subcluster were extracted from Data S1 l.

To evaluate the impact of EC-specific *Ihh* deletion on choroid immune cells, we first sought to identify the different cell types within choroidal hematopoietic cells and to characterize their molecular identity. We reanalyzed our scRNAseq data using only cells from cluster 11 (Fig. 1 b), and unsupervised cell clustering resulted in five transcriptionally distinct subclusters (Fig. 6 a and Data S1 l). We assembled lists of specifically enriched genes (average normalized UMIs at least fivefold higher compared with other hematopoietic subclusters; adj P < 0.05; Data S1 m), which were used to assess the identity of each subcluster using the Immunological Genome Project website (Heng et al., 2008; http://www.immgen.org; Fig. S4 a). Such analysis allowed us to identify them as macrophages (subcluster H2), T cells (H3), dendritic cells (DCs; H4), and mast cells (H5). The identity of subcluster H1 was not obvious, since some H1-specific genes were found to be expressed in DCs, macrophages, monocytes, and neutrophils (Fig. S4 a). After close inspection of our scRNAseq data, we classified H1 as *Itgae* (CD103)^−^ *Itgam* (CD11b)^+^ classical DCs (cDCs) and H4 as CD103^+^ CD11b^−^ cDCs (Merad et al., 2013; Fig. S4 b).

Next, we analyzed how EC-specific *Ihh* deletion modulates choroidal immune cells in vivo by combining our scRNAseq characterization of choroidal cell types and our bulk RNAseq analyses of RPE/choroid from *Ihh^iΔEC/iΔEC^* mice. We selected genes specifically enriched in hematopoietic cells (cluster 11 in Fig. 1 b; average normalized UMIs at least fivefold higher compared with other cell types; adj P < 0.05; Data S1 c) that were significantly reduced after *Ihh* deletion (adj P < 0.05; Data S1 h). To obtain more hits than those shown in Fig. 4 b (*Cma1* and *Gpr34*), we did not apply any FPKM or fold-change threshold to bulk RNAseq data. This analysis revealed that, in addition to *Cma1* and *Gpr34*, RPE/choroid expression of the hematopoietic-enriched genes *Cd163*, *Pf4* (CXCL4), *Mrc1* (CD206), and *Cd72* was significantly reduced after EC-specific *Ihh* deletion (Fig. 6 b). With the exception of *Cma1* (specifically expressed in mast cells, as expected), the rest of hematopoietic-enriched genes that were down-regulated in RPE/choroid tissue from *Ihh^iΔEC/iΔEC^* mice were markedly enriched in the macrophage population (Fig. 6 c). Interestingly, *Cd163* and *Mrc1* (CD206) are well-established markers of alternatively activated M2 macrophages, which are generally involved in inflammation resolution and tissue remodeling (Biswas and Mantovani, 2010; Murray, 2017). We performed immunofluorescence assays and quantitative imaging analyses on RPE/choroid flatmounts from albino *Ihh^+/+^* and *Ihh^iΔEC/iΔEC^* mice using antibodies against the murine pan-macrophage marker F4/80 and the M2 marker CD206. EC-specific *Ihh* deletion did not alter the percentage of F4/80^+^ area, suggesting the presence of a similar number of macrophages than in control animals (Fig. 6, d and e). However, *Ihh^iΔEC/iΔEC^* mice showed reduced CD206 expression in F4/80^+^ cells (Fig. 6, d and f). In summary, our observations suggest that (1) the main choroidal hematopoietic cell types affected by EC-specific *Ihh* deletion are mast cells and macrophages and (2) EC-specific *Ihh* deletion skews macrophage polarization away from the anti-inflammatory M2 phenotype.

In light of these results, we reasoned that inducing RPE/choroid injury in *Ihh^iΔEC/iΔEC^* mice might result in a longer or more pronounced inflammatory response than in control animals. To test this hypothesis, we performed experiments based on NaIO_3_ administration, a well-established model of retinal injury that induces selective RPE death in a dose-dependent manner and subsequent photoreceptor loss and vision impairment (Kannan and Hinton, 2014). We injected i.v. a single, low dose (15 mg kg^−1^) of NaIO_3_ into *Ihh^+/+^* and *Ihh^iΔEC/iΔEC^* mice, and 3 d later, RNA was extracted from RPE/choroid and neural retina to assess the expression of proinflammatory markers by real-time PCR. We observed a trend toward increased expression of all genes tested in both RPE/choroid and neural retina from *Ihh^iΔEC/iΔEC^* mice, reaching statistical significance for *Ccl2* and *Tnf* in both tissues and *Il6* and *Cxcl10* in neural retina (Fig. 6 g). To test the potential functional consequences of such augmented proinflammatory profile, we performed optomotor tracking assays to assess visual function in *Ihh^+/+^* and *Ihh^iΔEC/iΔEC^* mice before and 3 d after NaIO_3_ administration. In the absence of NaIO_3_ treatment, EC-specific *Ihh* deletion did not compromise visual function (Fig. 6 h). However, NaIO_3_ treatment induced a significantly impaired spatial visual function of *Ihh^iΔEC/iΔEC^* mice compared with *Ihh^+/+^* mice. These results demonstrate that in a context of tissue damage, EC-specific *Ihh* deletion results in an exacerbated inflammatory response in the RPE/choroid and neural retina that correlates with a more severe visual function impairment.

## Discussion

Herein, we report the transcriptome of mouse RPE/choroid tissue at single cell resolution and a study of HH signaling in the adult choroid. Taken together, our results support a model in which choroidal EC-expressed *Ihh* and HH-responding MSC-like cells are key for several aspects of choroidal and retinal immune homeostasis, including survival of choroidal mast cells and inflammatory response after tissue damage. Our findings constitute a new angle for the study of inflammation-related ocular disorders such as AMD.

A main contribution of our work is the identification and molecular characterization of transcriptionally distinct subtypes of choroidal ECs. As increasing evidence indicates that microvascular ECs are tissue specific and secrete specialized sets of angiocrine factors that regulate organ homeostasis and regeneration (Rafii et al., 2016), it was intriguing to find that choriocapillaris ECs, located next to RPE cells, exhibit a highly specific transcriptional profile, including a marked enrichment in *Ihh* expression. Previous work in the developing mouse eye has shown that *Ihh* is expressed by a subset of choroidal cells adjacent to the RPE presumed to be ECs based on the expression of collagen IV (Dakubo et al., 2003, 2008; Wallace and Raff, 1999). However, whether *Ihh* expression is maintained in the adult choroid remained unknown so far. Our scRNAseq and bulk RNAseq analyses unambiguously demonstrate that *Ihh* is expressed at high levels in adult mouse choriocapillaris ECs relative to other ECs in the choroid, retina, and extraocular tissues. The abovementioned study showed that *Ihh* knockout mice (which die during embryonic stages or at birth; St-Jacques et al., 1999) display defects in RPE, sclera, and neural retina prenatal development (Dakubo et al., 2008); now, we provide evidence that *Ihh* expression also plays an important role in adult choroid homeostasis.

Experiments using reporter *Gli1^GFP/+^* mice revealed that the main target of choroidal HH signaling is a population of perivascular cells that molecular, cellular, and functional assays identified as GLI1^+^ MSC-like stromal cells, in agreement with previous findings in other organs (Kramann et al., 2015; Zhao et al., 2014). Importantly, response of these cells to HH signaling seems to regulate the homeostasis of choroidal mast cells and melanocytes. It remains to be determined the role of this signaling circuit in the human choroid and its potential disease-associated alterations; nevertheless, the well-established immunomodulatory ability of MSCs (Uccelli et al., 2008) and the involvement of inflammation in several choroid-related eye pathologies such as AMD, diabetic retinopathy, and uveitis suggest that the choroid HH pathway we have uncovered may help in the understanding and treatment of such diseases. Interestingly, transcriptional analysis of RPE/choroid tissue from AMD donors and age-matched controls showed that expression of the proinflammatory genes *CCL2* and *CXCL10*, which is up-regulated upon tissue damage after EC-specific *Ihh* deletion (Fig. 6 g), is elevated in all major clinical AMD phenotypes (Newman et al., 2012). The role of HH signaling in the control and resolution of inflammation has been described in several pathological contexts, especially in the intestine, at the level of both HH ligands and target cells. In humans, a *GLI1* nonsynonymous polymorphism is strongly associated with ulcerative colitis, where areas of colonic inflammation display reduced expression of the HH target genes *GLI1*, *PTCH*, and *HHIP* (Lees et al., 2008). In mice, specific deletion of *Ihh* from enteric cells induces the expression of inflammation-related genes, leukocyte infiltration of the crypt area, and the development of intestinal fibrosis (van Dop et al., 2010; Westendorp et al., 2018). In agreement with our findings in the choroid, the main target of intestinal HH signaling is a population of fibroblast-like stromal cells (Westendorp et al., 2018). Moreover, inhibition of intestinal HH signaling by overexpression of the HH inhibitor HHIP induces spontaneous inflammation, diarrhea, weight loss, and death (Zacharias et al., 2010). Using a mouse model of chemically induced colitis, it was shown that inhibition or activation of the HH pathway intensifies or ameliorates colon inflammation, respectively (Lee et al., 2016). Interestingly, this study showed that HH activation induces *Il10* expression by stromal cells and results in increased numbers of intestinal CD4^+^FOXP3^+^ regulatory T cells. Given the connection between regulatory T cells and mast cells (see below), it would be interesting to study whether in the intestine, similarly to our observations in the choroid, HH signaling regulates the presence of mast cells. Importantly, intestinal mesenchyme responds to HH signaling in vitro by down-regulating proinflammatory genes (Zacharias et al., 2010), in line with our findings using cultured choroidal GLI1^+^ cells. Furthermore, the HH pathway acts as an endogenous anti-inflammatory system at the level of the blood–brain barrier (Alvarez et al., 2011). We now add the choroid to the list of adult tissues where the inflammatory response is modulated by HH signaling.

Additional in vivo and in vitro experiments indicated that HH-stimulated MSC-like cells are key for choroidal mast cell homeostasis and survival. Although mast cells are present in the choroid of humans and most vertebrates (May, 1999; McMenamin, 1997; McMenamin and Polla, 2013), their role in the eye in health and disease remains unknown. AMD is associated with an increased number of total and degranulated mast cells in the choroid (Bhutto et al., 2016; McLeod et al., 2017). However, the interpretation of these observations is unclear, as mast cells not only participate in proinflammatory responses but also can limit inflammation or facilitate its resolution (Caughey, 2011; Galli et al., 2008; Tsai et al., 2011). Mast cells are key for the immunosuppressive effect of UVB radiation (Hart et al., 1998) and the induction of tolerance to skin allografts mediated by CD4^+^FOXP3^+^ regulatory T cells (Lu et al., 2006). Interestingly, genetic mast cell depletion results in increased numbers of infiltrating immune cells in the eye after endotoxin-induced uveitis, suggesting that choroidal mast cells may play a protective, anti-inflammatory role in this organ (Smith et al., 1998). Indeed, our scRNAseq data show that mast cells are the only choroidal cell type with detectable expression of *Il4* (Data S1, b and m). Given the well-established role of IL-4 in promoting macrophage polarization toward the anti-inflammatory M2 phenotype (Biswas and Mantovani, 2010; Murray, 2017), it is possible that mast cell loss contributes to the reduction in M2 markers we observed in choroidal macrophages from *Ihh^iΔEC/iΔEC^* mice (Fig. 6, c and d–f). Hence, our results suggest that impairment of HH signaling may impact the pathogenesis of inflammatory choroid diseases through a decrease in the population of choroidal mast cells.

Because RPE and photoreceptor death is a hallmark of AMD, significant progress has been made to generate stem cell–derived RPE and photoreceptors for transplantation purposes (Nazari et al., 2015). However, as choriocapillaris loss is an early event during the development of AMD (Bhutto and Lutty, 2012; Whitmore et al., 2015), and because oxygen and nutrient supply of the outer retina depends on choroidal perfusion, it is likely that long-lasting engraftment and proper function of transplanted retinal cells could be significantly improved by cotransplantation of choriocapillaris ECs. However, the generation of such specialized ECs is challenging, in part due to the lack of information about their molecular identity. To date, only one laboratory has reported the generation of stem cell–derived choroid EC-like cells, and the assessment of their resemblance to authentic choriocapillaris ECs was limited to the expression of a few markers (Songstad et al., 2015, 2017). Although our characterization of choroidal EC subtypes by scRNAseq needs further validation in human tissue, it demonstrates marked gene expression differences between the choriocapillaris and other choroidal endothelial layers. We believe such transcriptome heterogeneity should be taken into account when generating stem cell–derived choriocapillaris ECs suitable for cell replacement strategies. Moreover, cell surface proteins encoded by choroidal EC signature genes (Fig. 2 c) constitute potential targets for site-directed delivery of functionalized nanoparticles or viral vectors after systemic administration, which could open new therapeutic approaches to treat choroidal and retinal pathologies.

## Materials and methods

### scRNAseq of mouse RPE/choroid tissue

Experiments were performed with 90-d-old mice of two different strains, C57BL/6J and B6129PF1/J. To isolate viable, single cells from RPE/choroid tissue, we euthanized the mice and washed the blood via intracardial perfusion with PBS buffer, and then enucleated the eyes for further processing. Each eye was cleaned from extraocular tissue and a circumpherencial incision was performed right below the ora serrata to remove the anterior segment, including cornea, lens, iris and ciliary body. The neural retina was detached from the optic nerve and RPE/choroid tissue was gently scraped from the sclera. For each strain, experiments included one eye from a male and another from a female, which were mixed as a strategy to estimate the percentage of contaminating doublets in our preparation. RPE/choroid tissue was incubated with Collagenase A (6.25 mg ml^−1^), Dispase II (6.25 mg ml^−1^), and DNase (62.5 µg ml^−1^; Roche) at 37°C for 15 min as previously described (Benedicto et al., 2017) and then in 0.25% trypsin-EDTA (Gibco) at 37°C for 5 min to create a single cell suspension. Both solutions also included Hoechst (10 µM; Thermo Fisher Scientific) to label nuclei during digestion. The cell suspension was pelleted, resuspended in sorting buffer (PBS plus 5% FBS and 2 mM EDTA), filtered through a 70-µm cell strainer, stained with TO-PRO-3 (1 µg ml^−1^; Thermo Fisher Scientific), and purified by FACS with gates set to include nucleated particles (Hoechst^+^) and exclude dead cells (TO-PRO-3^+^).

Library preparation, sequencing, and raw data after processing were performed at the Weill Cornell Medicine Epigenomics Core. scRNAseq libraries were prepared according to 10x Genomics specifications (Single Cell 3′ Reagent Kits v2 User Guide PN-120236; 10x Genomics). Cellular suspensions at a concentration between 250 and 500 cells/µl were loaded onto the 10x Genomics Chromium platform to generate barcoded single-cell gel bead-in-emulsion (GEMs), targeting ∼5,000 single cells per sample. GEM-reverse transcription (53°C for 45 min, 85°C for 5 min) was performed in a C1000 Touch thermal cycler with 96-Deep Well Reaction Module (Bio-Rad). Next, GEMs were broken and the single-strand cDNA was cleaned up with DynaBeads MyOne Silane Beads (Thermo Fisher Scientific). The cDNA was amplified (98°C for 3 min; 98°C for 15 s, 67°C for 20 s; and 72°C for 1 min × 12 cycles) using the C1000 Touch Thermal cycler with 96-Deep Well Reaction Module. Quality of the cDNA was assessed using an Agilent Bioanalyzer 2100, obtaining a product of ∼1,170 bp. Amplified cDNA was enzymatically fragmented, end repaired, A-tailed, subjected to a double-sided size selection with SPRIselect beads (Beckman Coulter), and ligated to adaptors provided in the kit. A unique sample index for each library was introduced through PCR amplification using the indexes provided in the kit (98°C for 45 s; 98°C for 20 s, 54°C for 30 s, and 72°C for 20 s × 14 cycles; and 72°C for 1 min). Indexed libraries were subjected to a second double-sided size selection, and libraries were then quantified using Qubit fluorometric quantification (Thermo Fisher Scientific). The quality was assessed on an Agilent Bioanalyzer 2100, obtaining an average library size of 455 bp.

Libraries were diluted to 2 nM and clustered on an Illumina HiSeq2500 high-output mode at 10 pM on a pair-end read flow cell and sequenced for 26 cycles on R1 (10x barcode and UMIs), followed by 8 cycles of I7 Index (sample Index), and 98 bases on R2 (transcript), with a coverage ∼145 million reads per sample. Primary processing of sequencing images was done using Illumina’s Real-Time Analysis software. 10x Genomics Cell Ranger Single Cell Software suite v2.1.0 was used to perform sample demultiplexing, alignment (mm10), filtering, UMI counting, single-cell 3′ end gene counting, and quality control using the manufacturer parameters. 3,996 (C57BL/6J mice) and 3,727 (B6129PF1/J mice) single cells were sequenced with ∼34,000 mean reads (∼75% sequencing saturation) and a median of ∼1,500 detected genes per cell.

Unsupervised cell clustering analysis was performed using the Seurat 2.1 R package (Butler et al., 2018). Cells with <1,700 UMIs, <600 genes, or >10% mitochondrial genes were excluded from the analysis, as well as genes detected in less than three cells. Gene expression raw counts were normalized following a global-scaling normalization method with a scale factor of 10,000 and a log transformation using the Seurat NormalizeData function. The top 2,000 highly variable genes from C57BL/6J and B6129PF1/J datasets were selected, followed by a canonical correlation analysis (CCA) to identify common sources of variation between the two datasets and minimize batch effect. The first 11 aligned CCA results were used to generate two-dimensional tSNE (RunTSNE in Seurat, perplexity = 30; Van der Maaten, 2008) and unsupervised cell clustering by a shared nearest neighbor (FindClusters in Seurat, k.param = 30 and resolution = 0.8). A list of conserved cell-type–specific genes were generated by FindConservedMarkers (test.use = “bimod”, logfc.threshold = 0.5, min.pct = 0.25) function in Seurat, identifying ≥65 conserved cell-type–specific genes for each cluster in both mouse strains (adj P < 0.05). Cells contained in cluster 11 (hematopoietic cells) were further subjected to a second round of unsupervised analysis following the same approach using the Seurat FindClusters with ∼1.3 resolution. The modified Seurat function FindAllMarkers was used to identify differentially expressed genes among subclusters within endothelial and hematopoietic cells. P values were calculated using likelihood-ratio test for single-cell gene expression (McDavid et al., 2013) and adjusted by the Benjamini–Hochberg method. More detailed arguments are available on Github (see Code Availability section).

Estimation of doublets was calculated based on the exclusive expression of *Xist* and *Ddx3y* in female and male cells, respectively. Expression of both genes associated to the same cellular barcode indicates the presence of a doublet. We made the following assumptions: (1) because we pooled the same amount of tissue from each sex before RPE/choroid digestion, 50% cells are male and 50% cells are female; (2) female–female and male–male doublet rates are the same as the female–male doublet rate; and (3) female–male doublets account for 50% of total doublets, since the other 50% are female–female and male–male doublets. We calculated *Xist* capture rate using the following equations:

$$Xist^{+}\mathrm{cells}=\left[\left(Xist^{+}Ddx3y^{-}\mathrm{cells}\right)+\left(Xist^{+}Ddx3y^{+}\mathrm{cells}\right)\right]$$

$$Xist^{+}\mathrm{capture}\;\mathrm{rate}=\left(Xist^{+}\;\mathrm{cells}/\mathrm{all}\;\mathrm{cells}\right)\times 2.$$

Note that the capture rate result was multiplied by 2 because only 50% of all cells are expected to be female. Similar equations were used to calculate *Ddx3y* capture rate:

$$Ddx3y^{+}\mathrm{cells}=\left[\left(Xist^{-}Ddx3y^{+}\mathrm{cells}\right)+\left(Xist^{+}Ddx3y^{+}\mathrm{cells}\right)\right]$$

$$Ddx3y^{+}\mathrm{capture}\;\mathrm{rate}=\left(Ddx3y^{+}\mathrm{cells}/\mathrm{all}\;\mathrm{cells}\right)\times 2.$$

Female–male doublet rate was calculated using the following equation:

$$\begin{array}{l} \left(\mathrm{All cells}\right)\times \left(Xist^{+}\mathrm{capture}\;\mathrm{rate}\right)\times \left(Ddx3y^{+}\mathrm{capture}\;\mathrm{rate}\right)\\ \times \left(\mathrm{female-male}\;\mathrm{doublet rate}\right)=Xist^{+}Ddx3y^{+}\mathrm{cells}. \end{array}$$

Because female–male doublet rate accounts for 50% of total doublets, the corrected doublet rate results from multiplying by 2 the female–male doublet rate. Calculations are shown on Fig. S1 b.

### Code availability

Custom code has been deposited at GitHub (https://github.com/nyuhuyang/scRNAseq-MouseEyes).

### Immunofluorescence assays and quantitative image analysis

For cryosection staining, mouse eyes were enucleated and transferred to 4% paraformaldehyde in PBS. A hole was made in the cornea with a 22G needle, and cornea and lens were removed. Eyecups were incubated in 4% paraformaldehyde for 2 h at room temperature. Eyes were washed in PBS, incubated in 15% sucrose in PBS for 30 min, and kept overnight at 4°C in 30% sucrose in PBS. Eyecups were infiltrated in Tissue-Tek optimal cutting temperature compound (Sakura) and frozen in the same solution at −80°C. Sections 5–10 µm thick were obtained with a cryostat (Hacker) and mounted on Superfrost Plus Gold Microscope Slides (Thermo Fisher Scientific). For immunostaining, eye sections were washed twice for 5 min in PBS, incubated for 1 h at room temperature in blocking buffer (PBS supplemented with 3% bovine serum albumin and 0.3% Triton X-100; Sigma) and incubated overnight at 4°C in blocking buffer plus the following primary antibodies: rat anti-mouse KDR (clone Avas12, 1:100, catalog number 136401; BioLegend), mouse anti-claudin 5 (clone 4C3C2, 1:500, catalog number 352588; Invitrogen), mouse anti-RPE65 (clone 401.8B11.3D9, 1:100, catalog number NB100-355; Novus Biologicals), rat anti-mouse CD140b (PDGFRβ; 1:200, catalog number 14–1402-81; Invitrogen), and/or rabbit anti-GFP (1:1,000, catalog number A11122; Invitrogen). After three washes in PBS for 10 min each, sections were incubated for 1 h at room temperature with Alexa Fluor 647 goat anti-rat and/or Alexa Fluor 488 goat anti-rabbit or anti-mouse antibodies (1:500; Life Technologies) in blocking buffer, washed three times in PBS for 10 min each, and mounted with Vectashield (Vector Laboratories). Intravital labeling of ECs was performed by retroorbital i.v. delivery of 20 µg Alexa Fluor 647–conjugated rat anti-mouse VE-cadherin (clone BV13, catalog number 138006; BioLegend) in mice previously anesthetized using isoflurane. After 10 min, mice were perfused with 4% paraformaldehyde and euthanized, and eyeballs were enucleated and processed for immunofluorescence as described above. To stain mast cells and macrophages in RPE/choroid flatmounts from albino mice, animals were dark-adapted overnight for better detachment of retina from the RPE and euthanized the following day. Eyes were enucleated and fixed in 4% paraformaldehyde in PBS for 2 h at room temperature, and cornea and lens were removed during fixation. Eyecups were washed twice in PBS and retina was carefully removed. RPE/choroid tissue was processed and stained as described above using Rhodamine Avidin D, TMRITC (1:250, catalog number A-2002; Vector Laboratories) for mast cell detection (Tharp et al., 1985; Veerappan et al., 2008), or rat anti-mouse F4/80 (1:500, catalog number ab6640; Abcam) and rabbit anti-mouse CD206 (1:500, catalog number ab64693; Abcam) plus Alexa Fluor 647 goat anti-rat and Alexa Fluor 568 goat anti-rabbit for macrophage staining. Images were collected with a Zeiss Axio Observer spinning disk confocal microscope equipped with a Yokogawa scanner unit, Hamamatsu Evolve electron-multiplying charge-coupled device cameras (Photometrics), and either a 63× (Fig. 3 c) or 20× (for flatmounts) objective. Images from Fig. 1 e were acquired using a Zeiss Axio Observer LSM 880 confocal microscope with a Plan Apochromat 40×/numerical aperture 0.95 objective. Mast cell quantification was performed using the open-source software Ilastik, which implements a random forest supervised machine learning classifier (Berg et al., 2019). Briefly, an initial training was performed using a control flatmount to separate foreground objects (mast cells) from the background and then applied to the remaining samples. The segmented binary images were exported and further processed in ImageJ using fill holes and watershed functions and then quantified using the analyze**particles function. Mast cell numbers were normalized to the total area for each flatmount. Macrophage quantification was performed with ImageJ software. After background subtraction (20 pixels), F4/80 signal was thresholded (default parameters) and used to create a selection, which was normalized by total flatmount area to calculate F4/80^+^ area. The same F4/80^+^ selection was applied to the background-subtracted CD206 image. CD206 mean intensity was measured within the F4/80^+^ selected area without applying any threshold.

### Isolation and RNAseq of tissue-specific mouse ECs

Mouse tissue-specific ECs were isolated from 30-d-old B6129PF1/J mice by intravital staining of VE-cadherin followed by tissue digestion and cell sorting as previously described (Benedicto et al., 2017). Cells were lysed immediately after sorting and processed for RNAseq as previously described (Benedicto et al., 2017). RNAseq was performed from three independent isolations, with 7 animals (14 eyes) per isolation for RPE/choroid and neural retina, and one mouse per isolation for liver, lung, and heart. cDNA libraries were prepared with the TruSeq RNA Sample Preparation Kit (Illumina) and sequenced on an Illumina HiSeq2000 platform. Upon quality control using FastQC, raw reads were aligned to the mouse genome (mm10), downloaded via the UCSC genome browser (http://hgdownload-test.cse.ucsc.edu/goldenPath/mm10/bigZips/) using STAR_2.4.0f1 (Dobin et al., 2013) and SAMTOOLS v0.1.19 (Li et al., 2009) for sorting and indexing reads. Cufflinks (2.0.2; Trapnell et al., 2012) was used to get the expression values (FPKM). Log-transformed FPKM profiles were clustered using hierarchical clustering (hclust function in R language) with average linkage and one minus Spearman correlation as distance. To determine the genes differentially expressed between choroidal ECs and ECs from neural retina, lung, liver, and heart, statistical significance was calculated using DEseq2 bioconductor package in R, which uses read counts (htseq-count; Love et al., 2014). With a detection threshold set at ≥1 FPKM, genes expressed in choroidal ECs at levels at least fivefold higher compared with ECs from neural retina, lung, liver, and heart (Benjamini–Hochberg corrected adj P < 0.05) were considered choroidal EC signature genes.

### Gene expression analyses of mouse ECs and RPE/choroid tissue by real-time PCR

For tissue-specific native ECs, RNA was extracted immediately after sorting using TRI Reagent (Molecular Research Center) and the RNeasy Mini Kit (Qiagen) as previously described (Benedicto et al., 2017). For mouse RPE/choroid tissue, mice were euthanized, and eye globes were enucleated followed by careful excision of any extraocular tissue that remained attached to the sclera. The anterior segment was discarded, and after removal of the neural retina, RPE/choroid tissue was mechanically dissected from the sclera using a scalpel and immediately used for RNA extraction with the RNeasy Mini Kit (Qiagen). cDNA was prepared with the High Capacity cDNA Reverse Transcription Kit (Life Technologies), and real-time PCR was performed in a StepOnePlus Real-Time PCR System (Life Technologies) using SYBR Select Master Mix (Life Technologies) and the following mouse primer pairs: *Ihh* (NM_010544, 5′-ACG​TGC​ATT​GCT​CTG​TCA​AG-3′ and 5′-GTC​TCC​TGG​CTT​TAC​AGC​TG-3′), *Gli1* (NM_010296, 5′-CCC​AGC​TCG​CTC​CGC​AAA​CA-3′ and 5′-CTG​CTG​CGG​CAT​GGC​ACT​CT-3′), *Cma1* (NM_010780, 5′-CAA​GCC​TGC​AAA​CAC​TTC​AC-3′ and 5′-CAT​AGG​ATG​CAA​TGC​CTT​GG-3′), *Mcpt4* (NM_010779, 5′-ATC​TGG​AGA​TCA​CCA​CTG​AG-3′ and 5′-TCA​CAT​CAT​GAG​CTC​CAA​GG-3′), *Tpsb2* (NM_010781, 5′-CCA​TTG​TCC​ATG​ATG​GCA​TG-3′ and 5′-TGT​AGA​TGC​CAG​GCT​TGT​TG-3′), *Fabp4* (NM_024406, 5′-GTG​ATG​CCT​TTG​TGG​GAA​CC-3 and 5′-TCA​TGT​TGG​GCT​TGG​CCA​TG-3′), *Spp1* (NM_001204233, 5′-TGC​CTG​ACC​CAT​CTC​AGA​AG-3 and 5′-TCG​TCG​TCC​ATG​TGG​TCA​TG-3′), *Il6* (NM_031168, 5′-GAA​CAA​CGA​TGA​TGC​ACT​TGC-3′ and 5′-TCC​AGG​TAG​CTA​TGG​TAC​TC-3′), *Ccl2* (NM_011333, 5′-CAT​TCA​CCA​GCA​AGA​TGA​TCC-3 and 5′-TGT​ATG​TCT​GGA​CCC​ATT​CC-3′), *Nos2* (NM_001313921, 5′-TGC​CTC​ATG​CCA​TTG​AGT​TC-3 and 5′-AGA​TGA​GCT​CAT​CCA​GAG​TG-3′), *Tnf* (NM_013693, 5′-CTC​TTC​TCA​TTC​CTG​CTT​GTG-3 and 5′-TCT​GGG​CCA​TAG​AAC​TGA​TG-3′), and *Cxcl10* (NM_021274, 5′-AGT​GAG​AAT​GAG​GGC​CAT​AG-3 and 5′-TCC​GGA​TTC​AGA​CAT​CTC​TG-3′). *Gapdh* (NM_008084, 5′-AAA​TGG​TGA​AGG​TCG​GTG​TG-3′ and 5′-GAG​TGG​AGT​CAT​ACT​GGA​AC-3′) was used as loading control, and relative expression values were calculated by the 2^−ΔΔCt^ method.

### Assessment of *IHH* expression in human tissues

Human retina and combined RPE/choroid punches were selected from a research collection at the University of Iowa Institute for Vision Research. This collection contains tissues obtained from the Iowa Lions Eye Bank (Iowa City, IA) after full consent of the donors’ families. All experiments conformed to the Declaration of Helsinki. The eyes used in this study had no known ocular history of AMD. Eyes were dissected by making a circumferential incision behind the limbus and removing the anterior segment consisting of cornea, iris, lens, and ciliary body. The remaining posterior pole was cut into a clover-leaf shape by making four equally spaced incisions through the sclera, choroid, RPE, and retina. An 8-mm sterile disposable trephine punch, centered on the fovea centralis, was collected from the macular region (easily identified by the presence of foveal pigment, proximity to the optic nerve head, and location between the superior and inferior arcades in the temporal retina). Neural retina and RPE/choroid samples were flash frozen separately in liquid nitrogen, and all procedures were completed within 5.5 h after death. Punches were stored at −80°C until used for RNA isolation. Samples were removed from the −80°C freezer and placed immediately on dry ice, such that they thawed in the presence of RNA extraction buffer (as even brief thawing is extremely destructive to RNA yield). Total RNA from RPE/choroid and neural retina was extracted using the RNeasy Mini Kit (Qiagen). Alternatively, total RNA from human lung, liver, and heart was obtained from Takara. Reverse transcription was performed in a 50-µl reaction using a reverse transcription kit (MultiScribe; Thermo Fisher Scientific). *IHH* expression was determined by real-time PCR (NM_002181, 5′-ATG​ACC​CAG​CGC​TGC​AAG-3′ and 5′-CAA​AGC​CGG​CCT​CCA​CTG-3′) as described previously (Zeng et al., 2012) using *RPL19* expression for normalization (NM_000981, 5′-ATG​CCA​ACT​CCC​GTC​AGC-3′ and 5′-ACC​CTT​CCG​CTT​ACC​TAT​GC-3′) and calculating relative expression values by the 2^−ΔΔCt^ method. All samples were analyzed in technical triplicates.

### Characterization of GLI1^+^ cells by flow cytometry

*Gli1^GFP/+^* reporter mice in which GFP expression is controlled by the endogenous *Gli1* promoter are described elsewhere (Brownell et al., 2011). Because these animals were generated in the Swiss strain (SWR/J), they present retinal degeneration due to the homozygous *rd1* mutation in the *Pde6b* gene (Chang et al., 2002). To avoid having confounding results due to retinal degeneration, we outcrossed these animals with wild-type BALB/c mice and intercrossed the offspring for at least two generations to remove the *rd1* phenotype before further characterization. RPE/choroid tissue from *Gli1^GFP/+^* mice was mechanically dissected from the sclera as described above and digested in collagenase A, dispase II and DNase as previously reported (Benedicto et al., 2017). Lungs were digested in the same conditions and used to compare the percentage of GFP^+^ cells. For staining, cells from two eyes were pelleted at 400 *g* for 5 min and resuspended in FACS buffer (PBS, 5% FBS, and 2 mM EDTA) including a CD16/CD32 antibody to block Fc receptors (clone 93, 1:200, catalog number 101302, BioLegend). Cells were filtered through a 40-µm cell strainer and incubated for 30 min at room temperature with the following fluorochrome-conjugated antibodies (BioLegend): CD45-APC/Cy7 (1:200, catalog number 103115), CD31-BV605 (1:200, catalog number 102427), CD29-PE/Cy7 (1:800, catalog number 102221), CD105-PerCP/Cy5.5 (1:800, catalog number 120415), PDGFRβ-PE (1:200, catalog number 136005), and SCA1-BV711 (1:200, catalog number 108131). Cells were washed three times in FACS buffer and resuspended in FACS buffer with 1 µg/ml DAPI to stain dead cells and exclude them from the analysis. Compensation controls were prepared using antibody-stained UltraComp eBeads (Thermo Fisher Scientific). Cells and beads were analyzed using a BD Fortessa flow cytometer and results were analyzed with FlowJo software.

### Generation of EC-specific *Ihh* knockout mice

Generation of EC-specific *Ihh* inducible knockout was achieved by breeding *Ihh^loxP/loxP^* mice (Jackson Laboratories) with *Cdh5*-Cre^ERT2^ transgenic mice (Wang et al., 2010) to establish the *Cdh5*-Cre^ERT2^ *Ihh^loxP/loxP^* line (*Ihh^iΔEC/iΔEC^*). To induce Cre activity, animals were i.p. treated with tamoxifen at a dose of 250 mg kg^−1^ in sunflower oil for 6 d (interrupted for 3 d after the third dose). After 8–12 wk of tamoxifen treatment, RPE/choroid tissue was processed for RNAseq, real-time PCR, or imaging assays. Albino *Ihh^+/+^* and *Ihh^iΔEC/iΔEC^* were generated by crossing pigmented *Ihh^iΔEC/iΔEC^* mice with albino *Gli1^+/+^* mice already free of *rd1* mutation (see above). Heterozygous agouti (brown color) *Ihh^+/iΔEC^* mice were obtained and crossed to generate albino homozygous *Ihh^iΔEC/iΔEC^* mice. Second-generation animals were used for either experiments or further breeding. In all experiments, tamoxifen-injected littermate *Cdh5*-Cre^ERT2^ *Ihh^+/+^* mice were used as controls, and both male and female animals were used.

### Culture, differentiation, and RNAseq analysis of choroidal GLI1^+^ cells

Sorting experiments to isolate *Gli1^+^* cells were performed at the Weill Cornell Medicine Flow Cytometry Core. RPE/choroid tissue from six *Gli1^GFP/+^* mice (female, 72 d old) was dissected and digested in collagenase A, dispase II, and DNase as described above. GFP^+^ live cells (DAPI^−^) were isolated with a BD Influx cell sorter into a well of a p24 plate containing 1 ml GLI1^+^ medium containing MEM α modification (Sigma) plus penicillin/streptomycin 50× solution (Corning), Glutamax 100× solution, and 20% FBS (Life Technologies). Cells were expanded up to three passages and frozen until further use.

For differentiation experiments, cells were cultured in StemXVivo Osteogenic/Adipogenic Base Media supplemented with either 100× adipogenic or 20× osteogenic supplement (R&D Systems) following the manufacturer’s instructions. After 21 d of differentiation, cells were processed for real-time PCR to assess the expression of adipogenic (*Fabp4*) and osteogenic (*Spp1*) markers (see above). Adipogenesis and osteogenesis were also monitored by staining with Oil Red O and Alizarin Red S (Sigma), respectively. At day 21, cells were washed in PBS and fixed in 4% paraformaldehyde for 30 min at room temperature. For adipogenesis assays, cells were incubated in 60% isopropanol for 5 min at room temperature, followed by a 15 min incubation in a filtered 3:2 dilution of Oil Red O stock solution (0.3% in isopropanol) and water. Cells were then washed with distilled water and imaged with an inverted microscope (Zeiss Axio Observer). For osteogenesis experiments, fixed cells were stained with 2% Alizarin Red S in water (pH 4.2) for 45 min at room temperature, extensively washed with water and photographed.

For RNAseq experiments, cultured GLI1*^+^* cells were starved overnight in 0.5% FBS−containing GLI1^+^ medium, followed by stimulation with 100 nM SAG (Cayman) or vehicle (DMSO). After a 24-h incubation, RNA from three biological replicates was extracted with the RNeasy Mini Kit (Qiagen) after direct lysis of cells in the culture plate and processed for RNAseq. Bioinformatics analyses were performed as described above. With a detection threshold set at ≥1 average FPKM in the condition with most abundant expression, genes whose expression changed at least twofold after SAG treatment (Benjamini–Hochberg corrected adj P < 0.05) were selected to carry out Gene Ontology analyses with DAVID software (Huang et al., 2009). Ingenuity Pathway Analysis (Qiagen) was performed with the same gene set but without FPKM or fold-change threshold. GSEAs (Subramanian et al., 2005) using the terms “immune response” (GO: 0006955), “cellular response to interferon-β” (GO: 0035458), and “cellular response to interferon-gamma” (GO: 0071346) were performed to compare the transcriptomes of control and SAG-treated cells.

### Mast cell survival assay

Confluent GLI1^+^ cells grown in 48-well plates were starved in 0.5% FBS-containing GLI1^+^ medium for 16 h, followed by stimulation with 100 nM SAG (Cayman) or vehicle (DMSO) for 24 h. As controls, we included DMSO- and SAG-containing wells without cells. RBL-2H3 cells (CRL-2256; ATCC) were labeled with CellTracker Red CMTPX dye (C34552; Thermo Fisher Scientific) according to the manufacturer’s instructions, and 25,000 cells/well were added on top of control or SAG-stimulated GLI1^+^ cells and on wells without GLI1^+^ cells, in the absence or presence of SAG. After 24 h, cultures were stained with DAPI (1:2,000, catalog number D21490; Invitrogen) for 2 min, and nonadherent cells were transferred to a new 48-well plate and centrifuged at 400 *g *for 5 min. Cells were fixed with 2% paraformaldehyde in PBS and images were obtained using an LSM510 confocal microscope using Zen software (Zeiss) in three 1,270 × 1,270 µm fields per well. Cell death was calculated as the percentage of CellTracker Red CMTPX dye–positive cells that were labeled with DAPI, using National Institutes of Health ImageJ software (version 1.49).

### Melanocyte proliferation assay in conditioned media from choroidal GLI1^+^ cells

To prepare conditioned media, GLI1^+^ cells were starved in 0.5% FBS-containing GLI1^+^ medium for 16 h, followed by stimulation with 100 nM SAG or vehicle (DMSO) for 48 h. As controls, we included DMSO- and SAG-containing wells without cells. Media was collected, cleared by centrifugation and used immediately. Melan-a cells were cultured in RPMI media as previously described (Bennett et al., 1987) and seeded in triplicate (2,500 cells/well) on black, clear-bottom 96-well plates in 100 µl culture media. After 24 h, cells were starved for further 24 h in 0.5% FBS-containing GLI1^+^ medium. Media was replaced with GLI1^+^ conditioned media prepared as described above. In parallel, we included melan-a cells seeded at the same density and grown in normal melan-a culture media that were representative of maximum growth. Cell number was measured 48 h after media change using the CyQUANT Direct Cell Proliferation Assay Kit (catalog number C35012; Thermo Fisher Scientific) and measuring fluorescence (480/535 nm excitation/emission) with a SpectraMax M2 Multi-Mode Microplane Reader (Molecular Devices). After background subtraction, relative cell number was calculated as the percentage of fluorescence intensity of melan-a cells cultured in their regular medium.

### T cell proliferation assays

The intracellular fluorescent dye CFSE (Invitrogen) was used to determine cell division in responder cells as previously described (Lyons et al., 2013). Briefly, splenocytes were obtained from BALB/c mice and washed using a 5-ml syringe to prepare a single-cell suspension. Red blood cells were lysed with ammonium chloride lysing buffer (0.15 M NHCl, 1 mM KHCO, and 0.1 mM EDTA) for 5 min at room temperature. Splenocytes were labeled with 1 µM CFSE in PBS for 7 min at 37°C. The reaction was stopped with ice-cold PBS supplemented with 10% FBS and washed twice. Labeled cells were cultured either alone or in co-culture at different ratios with GLI1^+^ cells in the presence of 1 µg ml^−1^ anti-CD3 monoclonal antibody (catalog number BE0001-1; Bio X Cell), which served as a cell activator. After 4 d of culture, responder nonadherent cells were harvested, stained with 0.5 µg ml^−1^ PerCP- and PE-labeled anti-CD4 and anti-CD8a antibodies, respectively (catalog numbers 553052 and 553033; BD PharMingen) and analyzed by flow cytometry (BD FACS CANTO II). CD4^+^ and CD8^+^ T cells were selected through gating and analyzed for CFSE fluorescence intensity. Data analysis was performed using FlowJo software (version 8; TreeStar) to obtain the division index (average number of cell divisions that a cell in the original population has undergone) and the percentage of cells from the original sample that divided.

### NaIO_3_ treatment and optomotor response assays

Visual thresholds of *Ihh^+/+^* and *Ihh^iΔEC/iΔEC^* mice were measured by evaluating optokinetic tracking in a virtual optokinetic system as described previously (Benedicto et al., 2017). After measuring baseline visual function, *Ihh^+/+^* and *Ihh^iΔEC/iΔEC^* mice were anesthetized and injected i.v. with a single dose of 15 mg kg^−1^ NaIO_3_ (Sigma) using a 30G needle. After 3 d, optomotor response assays were performed and mice were euthanized. Eyes were processed for RNA extraction from RPE/choroid and neural retina, and relative gene expression was assessed by real-time PCR (see above).

### Statistical analyses

All graphs show individual data points and their average. The number of biological replicates (*n*) is indicated in each figure legend. Statistical significance was calculated using unpaired two-tailed *t* test or one-way ANOVA plus Bonferroni post hoc analysis (*, P < 0.05; **, P < 0.01; and ***, P < 0.001) as indicated. No statistical method was used to predetermine sample size or to test for normality and variance homogeneity. Data points >1.5 interquartile ranges below the first quartile or above the third quartile were considered outliers and excluded from the analyses. All in vivo experiments were blinded and included littermate controls.

### Ethical compliance

All animal protocols were reviewed and approved by the Institutional Animal Care and Use Committee at Weill Cornell Medicine.

### Data availability

All data supporting the findings of this study are available within the article and its supplemental information files or upon request. Bulk RNAseq data from mouse choroid ECs (Benedicto et al., 2017) were previously deposited in the Gene Expression Omnibus under series number GSE95835. RPE/choroid scRNAseq and bulk RNAseq data (tissue-specific ECs, RPE/choroid from *Ihh^+/+^* and *Ihh^iΔEC/iΔEC^* mice, and cultured GLI1^+^ cells ± SAG) have been deposited in the Gene Expression Omnibus under series number GSE135167.

### Online supplemental material

Fig. S1 shows data regarding scRNAseq analyses, including the cell-sorting approach used to generate RPE/choroid single-cell suspensions, superimposed tSNE plots from C57BL/6J and B6129PF1/J mouse strains, calculations for doublet estimation, and cluster-specific gene expression for C57BL/6J mice. Fig. S2 shows the gating strategy used to analyze GLI1^+^ cells by flow cytometry. Fig. S3 shows loss of mast cells in *Gli1*-null mice, down-regulation of melanocyte markers in RPE/choroid tissue after EC-specific *Ihh* deletion, and increased melanocyte proliferation in vitro in the presence of conditioned media from SAG-stimulated GLI1^+^ cells. Fig. S4 shows the bioinformatics characterization of choroidal hematopoietic cells. Data S1 shows complete datasets derived from scRNAseq and bulk RNAseq analyses.

## Supplementary Material

Data S1 contains datasets derived from scRNAseq and bulk RNAseq analyses.
